# Microbial monoterpene transformations—a review

**DOI:** 10.3389/fmicb.2014.00346

**Published:** 2014-07-15

**Authors:** Robert Marmulla, Jens Harder

**Affiliations:** Department of Microbiology, Max Planck Institute for Marine MicrobiologyBremen, Germany

**Keywords:** isoprenoids, acyclic monoterpene utilization, camphor, pinene, limonene, linalool, myrcene, eucalyptol

## Abstract

Isoprene and monoterpenes constitute a significant fraction of new plant biomass. Emission rates into the atmosphere alone are estimated to be over 500 Tg per year. These natural hydrocarbons are mineralized annually in similar quantities. In the atmosphere, abiotic photochemical processes cause lifetimes of minutes to hours. Microorganisms encounter isoprene, monoterpenes, and other volatiles of plant origin while living in and on plants, in the soil and in aquatic habitats. Below toxic concentrations, the compounds can serve as carbon and energy source for aerobic and anaerobic microorganisms. Besides these catabolic reactions, transformations may occur as part of detoxification processes. Initial transformations of monoterpenes involve the introduction of functional groups, oxidation reactions, and molecular rearrangements catalyzed by various enzymes. *Pseudomonas* and *Rhodococcus* strains and members of the genera *Castellaniella* and *Thauera* have become model organisms for the elucidation of biochemical pathways. We review here the enzymes and their genes together with microorganisms known for a monoterpene metabolism, with a strong focus on microorganisms that are taxonomically validly described and currently available from culture collections. Metagenomes of microbiomes with a monoterpene-rich diet confirmed the ecological relevance of monoterpene metabolism and raised concerns on the quality of our insights based on the limited biochemical knowledge.

## Introduction

Annually about 120 Pg of carbon dioxide are assimilated by plants. A part is transformed into chemically complex molecules and released into the environment by emission or excretion (Ghirardo et al., 2011). Volatile organic compounds (VOCs) comprise a large number of molecules, including various hydrocarbons, single carbon compounds (e.g. methane), isoprene and terpenes (e.g. mono- and sesquiterpenes). The atmosphere is loaded with an estimated VOC emission rate of about 1150 Tg C yr^−1^ (Stotzky and Schenck, 1976; Guenther et al., 1995; Atkinson and Arey, 2003). These estimates included only non-methane VOCs of biogenic origin (BVOCs); a second source are anthropogenic VOCs. Among the BVOCs, isoprene and monoterpenes dominate with estimated emission rates of about 500 Tg C yr^−1^ and 127 Tg C yr^−1^, respectively (Guenther et al., 1995). Monoterpenes (C_10_H_16_) consist of two linked isoprene (C_5_H_8_) units and include in the strict sense only hydrocarbons. Often the term monoterpene is applied including monoterpenoids which are characterized by oxygen-containing functional groups. Structural isomers—acyclic, mono-, and bicyclic monoterpenes—, stereoisomers as well as a variety of substitutions result in a large diversity of molecules. Today, more than 55,000 different isoprenoids are known (Ajikumar et al., 2008). Monoterpenes are not only emitted as cooling substances (Sharkey et al., 2008), but can also be stored intracellularly serving mainly as deterrent or infochemical (Dudareva et al., 2013). Wood plants mainly accumulate pinene and other pure hydrocarbon monoterpenes as constituents of their resins, whereas citrus plants are the major source of limonene. Flowers, however, produce and emit a variety of oxygenated monoterpenes (e.g. linalool) (Kesselmeier and Staudt, 1999 and references therein, Sharkey and Yeh, 2001; Bicas et al., 2009).

In the atmosphere, monoterpenes are transformed in purely chemical reactions within hours. Photolysis and reactions with molecular oxygen, ozone, hydroxyl radicals, NO_x_ species, and chlorine atoms result in carbonyls, alcohols, esters, halogenated hydrocarbons, and peroxynitrates. These products condense and lead to the formation of secondary aerosols. Rain or precipitation transports them to soils (Atkinson and Arey, 2003; Fu et al., 2009; Ziemann and Atkinson, 2012). Monoterpenes reach the surface layers of soils by leaf fall and excreted resins. Also roots emit monoterpenes into the rhizosphere (Wilt et al., 1993; Kainulainen and Holopainen, 2002). Deeper soil layers do contain significant less monoterpenes than the surface soil layer. Emission into the atmosphere and biotransformations in the surface layer mainly by microorganisms are the major sinks. An alternative, abiotic photoreactions like in the atmosphere, is limited by light availability in soil (Kainulainen and Holopainen, 2002; Insam and Seewald, 2010).

**Figure 1:**
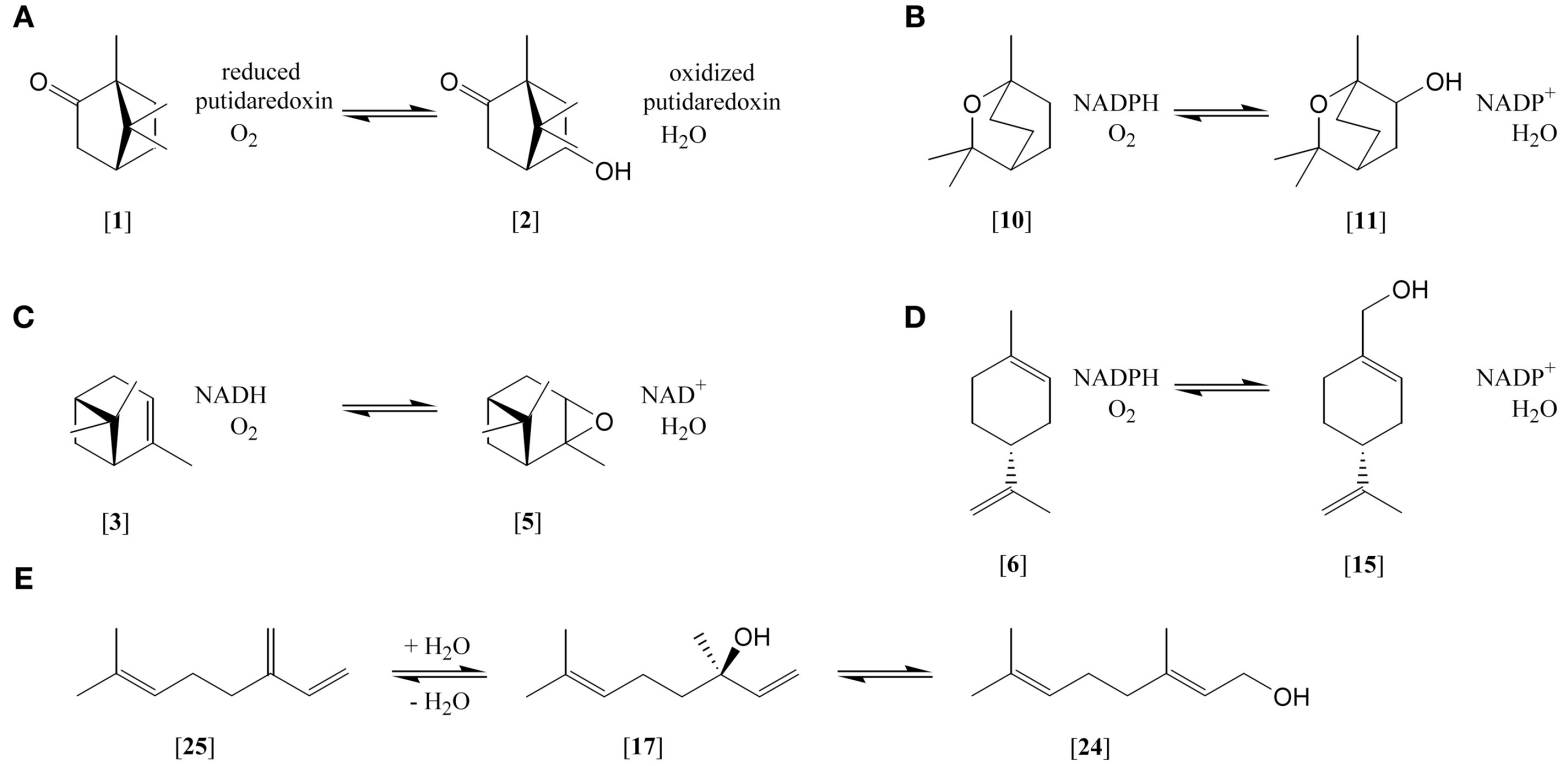
**Selected monoterpene transformations. (A)** (+)-camphor [1] hydroxlation to 5-hydroxycamphor [2]; **(B)** 1,8-cineole [10] hydroxylation to hydroxy-1,8-cineole [11]; **(C)** α-pinene [3] epoxidation to α-pinene oxide [5]; **(D)** (*R*)-limonene [6] hydroxylation to perillyl alcohol [15]; **(E)** myrcene [25] hydration to (*S*)-(+)-linalool [17] and isomerization to geraniol [24].

Bacteria encountering monoterpenes have to deal with their toxic effects (reviewed by Bakkali et al., 2008). In order to prevent the accumulation of monoterpenes in the cell and cytoplasmatic membrane, bacteria modify their membrane lipids, transform monoterpenes and use active transport by efflux pumps (Papadopoulos et al., 2008; Martinez et al., 2009). Below toxic concentrations monoterpenes are used by microorganisms as sole carbon and energy source. The mineralization of the hydrocarbons requires the introduction of functional groups to access beta-oxidation like fragmentation reactions yielding central metabolites, e.g. acetyl-CoA. In many aerobic microorganisms molecular oxygen serves as reactive agent to functionalize the monoterpenes (Figure 1). Strains of *Pseudomonas* and *Rhodococcus* have become model organisms for the elucidation of pathways in aerobic bacteria. Nearly 40 years after the first reports on aerobic mineralization (Seubert, 1960; Seubert and Fass, 1964; Dhavalikar and Bhattacharyya, 1966; Dhavalikar et al., 1966), the mineralization of monoterpenes in denitrifying bacteria and methanogenic communities was discovered (Harder and Probian, 1995; Harder and Foss, 1999). Betaproteobacterial strains of the genera *Castellaniella* and *Thauera* are the study objects for the elucidation of anaerobic pathways. All these bacteria were obtained in single-fed batch enrichments with high substrate concentrations (mmol^*^L^−1^), in contrast to low concentrations in nature (μmol^*^L^−1^). Consequently, in batch enrichments isolated strains exhibit often a solvent tolerance; they grow in the presence of a pure monoterpene phase. Cultivation was rarely attempted by physical separation followed by single-fed batch cultivations. Such dilution-to-extinction series performed in replicates—also known as most-probable-number (MPN) method—revealed a frequent presence of the degradative capacities in natural populations: denitrifying communities in sewage sludge and forest soil yielded 10^6^–10^7^ monoterpene-utilizing cells ml^−1^, representing 0.7–100% of the total cultivable nitrate-reducing microorganisms (Harder et al., 2000). MPN cultivations for aerobic bacteria have not been reported so far, and for both cases the highly abundant bacteria with the capacity to grow on monoterpenes have not been identified.

Over the last 50 years, many monoterpene transformations have been reported for microbial cultures, but the biochemical pathways were rarely disclosed. More important for the maintenance of our knowledge, only a small portion of the investigated strains were deposited in culture collections. Without detailed knowledge of genes or the availability of strains, the observations of biotransformation experiments are of limited value for future studies. Therefore, this review on the transformation of monoterpenes focusses on enzymes for which the gene and protein sequences are available in public databases as well as on microorganisms that at least have been deposited in a public culture collection and ideally are validly described (Table 1). A broad overview on microbial biotransformations is also provided by a number of older review articles (Trudgill, 1990, 1994; van der Werf et al., 1997; Hylemon and Harder, 1998; Duetz et al., 2003; Ishida, 2005; Li et al., 2006; Bicas et al., 2009; Li and Lan, 2011; Schewe et al., 2011; Tong, 2013). KEGG and MetaCyc, two widely used reference datasets of metabolic pathways (reviewed by Altman et al., 2013), include degradation pathways of limonene, pinene, geraniol, and citronellol. Single reactions of *p*-cymene and *p*-cumate degradation are covered. MetaCyc additionally covers the metabolism of myrcene, camphor, eucalyptol, and carveol.

**Table 1:** **Summary table of monoterpene transforming enzymes in validly described species of *Bacteria***.

**EC number**	**Enzyme name**	**Organism**	**Substrate**	**Co-substrate**	**Product**	**Co-product**	**References**
1.14.13.155	α-pinene monooxygenase	*Pseudomonas fluorescens* NCIMB 11671	α-pinene	Oxygen, NADH	α-pinene oxide	Water, NAD^+^	Best et al., 1987
5.5.1.10	α-pinene oxide lyase	*Pseudomonas fluorescens* NCIMB 11671	α-pinene oxide		(*E*)-2,6-dimethyl-5-methylidene-hept-2-enal (iso-novalal)		Best et al., 1987
		*Pseudomonas rhodesiae* PF1 (CIP 107491)					Fontanille et al., 2002
1.14.13.156	1,8-cineole 2-endo-monooxygenase	*Citrobacter braakii*	1,8-cineole	Oxygen, NADPH	2-*endo*-hydroxy-1,8-cineole	Water, NADP^+^	Hawkes et al., 2002
1.14.13.105	Monocyclic monoterpene ketone monooxygenase	*Rhodococcus erythropolis* DCL 14	(−)-menthone	Oxygen, NADPH	(4*R*,7*S*)-4-methyl-7-(propan-2-yl) oxepan-2-one	Water, NADP^+^	van der Werf et al., 1999b
1.1.1.297	Limonene 1,2-diol dehydrogenase	*Rhodococcus erythropolis* DCL 14	Limonene 1,2-diol	NAD^+^	1-hydroxy-*p*-menth-8-en-2-one	NADH	van der Werf et al., 1999b
1.14.13.107	Limonene 1,2-monooxygenase	*Rhodococcus erythropolis* DCL 14	(*R*)-limonene	Oxygen, NAD(P)H	1,2-epoxy-menth-8-ene	Water, NAD(P)^+^	van der Werf et al., 1999b
1.14.13.48	(*S*)-limonene 6-monooxygenase	*Rhodococcus erythropolis* DCL 14	(*S*)-limonene	Oxygen, NADPH	(−)-*trans*-carveol	Water, NADP^+^	van der Werf et al., 1999b
1.1.1.243	Carveol dehydrogenase	*Rhodococcus erythropolis* DCL 14	(−)-*trans*-carveol	NADP^+^	(−)-carvone	NADPH	van der Werf et al., 1999b
1.3.99.25	Carvone reductase	*Rhodococcus erythropolis* DCL 14	(+)-dihydrocarvone	Oxidized electron acceptor	(−)-carvone	Reduced electron acceptor	van der Werf et al., 1999b
1.1.1.275	*Trans*-carveol dehydrogenase	*Rhodococcus opacus* PWD4 (DSM 44313)	(+)-*trans*-carveol	NAD^+^	(+)-carvone	NADH	Duetz et al., 2001
3.1.1.83	Monoterpene ε-lactone hydrolase	*Rhodococcus erythropolis* DCL 14	(4*S*,7*R*)-7-methyl-4-prop-1-en-2-yl-oxepan-2-one	Water	6-hydroxy-3-prop-1-en-2-yl-heptanoate		van der Werf et al., 1999a
3.3.2.8	(4*R*)-limonene-1,2-epoxide hydrolase	*Rhodococcus erythropolis* DCL 14	1,2-epoxy-*p*-menth-8-ene	Water	Menth-8-ene-1,2-diol		van der Werf et al., 1999a
1.1.1.297	(1*S*,2*S*,4*R*)-limonene-1,2-diol dehydrogenase	*Rhodococcus erythropolis* DCL 14	Menth-8-ene-1,2-diol	NAD^+^	1-hydroxy-*p*-menth-8-en-2-one	NADH	van der Werf et al., 1999a
1.14.13.49	(*S*)-limonene 7-monooxygenase	*Geobacillus stearothermophilus (*ex *Bacillus* strain BR388*)*	(*S*)-limonene	Oxygen, NADPH	(−)-perillyl alcohol	Water, NADP^+^	Cheong and Oriel, 2000
1.14.13.151	Linalool 8-monooxgenase	*Novosphingobium aromaticivorans* ATCC 700278D-5	Linalool	2 oxygen, 2 NADH	(6*E*)-8-oxolinalool	3 Water, 2 NAD^+^	Bell et al., 2010
		*Pseudomonas putida* PpG777					Ullah et al., 1990
4.2.1.127	Linalool dehydratase (-isomerase)	*Castellaniella defragrans* 65Phen (DSM 12143)	β-myrcene	Water	(*S*)-(+)-linalool		Brodkorb et al., 2010
5.4.4.4	Linalool (dehydratase)-isomerase	*Castellaniella defragrans* 65Phen (DSM 12143)	(*S*)-(+)-linalool		Geraniol		Brodkorb et al., 2010
1.1.1.347	Geraniol dehydrogenase	*Castellaniella defragrans* 65Phen (DSM 12143)	Geraniol	NAD^+^	Geranial	NADH	Lueddeke et al., 2012
1.2.1.86	Geranial dehydrogenase	*Castellaniella defragrans* 65Phen (DSM 12143)	Geranial	Water, NAD^+^	Geranic acid	NADH	Lueddeke et al., 2012
**Cym PATHWAY**							
1.14.13.-	*p*-cymene monooxygenase, hydroxylase subunit (CymAa)	*Pseudomonas putida* F1 (ATCC 700007)	*p*-cymene	Oxygen, NADH	*p*-cumic alcohol	Water, NAD^+^	Eaton, 1997
1.14.13.-	*p*-cymene monooxygenase, reductase subunit (CymAb)	*Pseudomonas putida* F1 (ATCC 700007)	*p*-cymene	Oxygen, NADH	*p*-cumic alcohol	Water, NAD^+^	Eaton, 1997
1.1.1.-	*p*-cumic alcohol dehydrogenase (CymB)	*Pseudomonas putida* F1 (ATCC 700007)	*p*-cumic alcohol	NAD^+^	*p*-cumic aldehyde	NADH	Eaton, 1996
1.2.1.-	*p*-cumic aldehyde dehydrogenase (CymC)	*Pseudomonas putida* F1 (ATCC 700007)	*p*-cumic aldehyde	Water, NAD^+^	*p*-cumic acid	NADH	Eaton, 1996
-.-.-.-	Putative outer membrane protein, unknown function (CymD)	*Pseudomonas putida* F1 (ATCC 700007)					Eaton, 1996
6.2.1.1	Acetyl-CoA synthetase (CymE)	*Pseudomonas putida* F1 (ATCC 700007)	Acetate	CoA, ATP	Acetyl-CoA	Diphosphate, AMP	Eaton, 1996
**Cmt PATHWAY**							
1.14.12.-	*p*-cumate 2,3-dioxygenase (CmtAaAbAcAd)	*Pseudomonas putida* F1 (ATCC 700007)	*p*-cumate	Oxygen, NADH	*Cis*-2,3-dihydroxy-2,3-dihydro-*p*-cumate	NAD^+^	Eaton, 1996
1.3.1.58	2,3-dihydroxy-2,3-dihydro-*p*-cumate dehydrogenase (CmtB)	*Pseudomonas putida* F1 (ATCC 700007)	*Cis*-2,3-dihydroxy-2,3-dihydro-*p*-cumate	NAD^+^	2,3-dihydroxy-*p*-cumate	NADH	Eaton, 1996
1.13.11.-	2,3-dihydroxy-*p*-cumate dioxygenase (CmtC)	*Pseudomonas putida* F1 (ATCC 700007)	2,3-dihydroxy-*p*-cumate	Oxygen	2-hydroxy-3-carboxy-6-oxo-7-methylocta-2,4-dienoate		Eaton, 1996
4.1.1.-	2-hydroxy-3-carboxy-6-oxo-7-methylocta-2,4-dienoate decarboxylase (CmtD)	*Pseudomonas putida* F1 (ATCC 700007)	2-hydroxy-3-carboxy-6-oxo-7-methylocta-2,4-dienoate		2-hydroxy-6-oxo-7-methylocta-2,4-dienoate	Carbon dioxide	Eaton, 1996
3.7.1.-	2-hydroxy-6-oxo-7-methylocta-2,4-dienoate hydrolase (CmtE)	*Pseudomonas putida* F1 (ATCC 700007)	2-hydroxy-6-oxo-7-methylocta-2,4-dienoate	Water	2-hydroxypenta-2,4-dienoate	Isobutyrate	Eaton, 1996
4.2.1.80	2-hydroxypenta-2,4-dienoate hydratase (CmtF)	*Pseudomonas putida* MT-2 (ATCC 33015)	2-hydroxy-penta-2,4-dienoate	Water	2-oxo-4-hydroxy-pentanoate		Harayama et al., 1989
4.1.3.39	2-oxo-4-hydroxyvalerate aldolase (CmtG)	*Pseudomonas putida* PG (DSM 8368)	2-oxo-4-hydroxy-pentanoate		Acetaldehyde	Pyruvate	Platt et al., 1995
1.2.1.10	Acetaldehyde dehydrogenase (CmtH)	*Pseudomonas putida* PG (DSM 8368)	Acetaldehyde	NAD^+^, CoA	Acetyl-CoA	NADH	Platt et al., 1995
**Atu PATHWAY**							
1.1.99.-/1.2.99.-	Citronellol/citronellal dehydrogenase (AtuB; AtuG)	*Pseudomonas citronellolis* (ATCC 13674)	Citronellol/citronellal	Water, oxidized electron acceptor	Citronellal/citronellate	Reduced electron acceptor	Förster-Fromme et al., 2006
6.2.1.-	Putative citronellyl-CoA synthetase (AtuH)	*Pseudomonas citronellolis* (ATCC 13674)	Citronellate	CoA, ATP	Citronellyl-CoA	Diphosphate, AMP	Förster-Fromme et al., 2006
1.3.99.-	Putative citronellyl-CoA desaturase (AtuD)	*Pseudomonas citronellolis* (ATCC 13674)	Citronellyl-CoA	Oxidized electron acceptor	*Cis*-geranyl-CoA	Reduced electron acceptor	Förster-Fromme et al., 2006
6.4.1.5	Geranyl-CoA carboxylase, carboxylase alpha-subunit (AtuF)	*Pseudomonas citronellolis* (ATCC 13674)	*Cis*-geranyl-CoA	Bicarbonate, ATP	Isohexenyl-glutaconyl-CoA	ADP, phosphate	Förster-Fromme et al., 2006
6.4.1.5	Geranyl-CoA carboxylase, carboxylase beta-subunit (AtuC)	*Pseudomonas citronellolis* (ATCC 13674)	*Cis*-geranyl-CoA	Bicarbonate, ATP	Isohexenyl-glutaconyl-CoA	ADP, phosphate	Fall and Hector, 1977; Förster-Fromme et al., 2006
	AtuC, AtuF	*Pseudomonas mendocina* (ATCC 25411)					Cantwell et al., 1978
	AtuC, AtuF	*Pseudomonas aeruginosa* PAO1 (ATCC 15692)					Díaz-Pérez et al., 2004; Höschle et al., 2005
4.2.1.57	Isohexenyl-glutaconyl-CoA hydratase (AtuE)	*Pseudomonas citronellolis* (ATCC 13674)	Isohexenyl-glutaconyl-CoA	Water	3-hydroxy-3-isohexenyl-glutaryl-CoA		Förster-Fromme et al., 2006
	AtuE	*Pseudomonas aeruginosa* PAO1 (ATCC 15692)					Díaz-Pérez et al., 2004; Höschle et al., 2005
	AtuE	*Pseudomonas mendocina* (ATCC 25411)					Cantwell et al., 1978
4.1.3.26	3-hydroxy-3-iso-hexenyl-glutaryl-CoA:acetate lyase (LiuE)	*Pseudomonas citronellolis* (ATCC 13674)	3-hydroxy-3-isohexenyl-glutaryl-CoA		7-methyl-3-oxo-6-octenoyl-CoA	Acetate	Förster-Fromme et al., 2006; Chávez-Avilés et al., 2010
	LiuE	*Pseudomonas aerugenosa* PAO1 (ATCC 15692)					Chávez-Avilés et al., 2010
**Cam PATHWAY**							
							(Iwaki et al., 2013, and references therein)
1.14.15.1	Camphor 5-monooxygenase (CamABC)	*Pseudomonas putida* (ATCC 29607)	(+)(-)-camphor	Oxygen, reduced putidaredoxin	5-oxo-hydroxy-camphor	Water, oxidized putidaredoxin	Poulos et al., 1985
		*Novosphingobium aromaticivorans* (ATCC 700278D-5)					Bell et al., 2010
1.1.1.327	5-exo-hydroxycamphor dehydrogenase (CamD)	*Pseudomonas putida* (ATCC 17453)	5-oxo-hydroxy-camphor	NAD^+^	2,5-diketocamphane/3,6-diketocamphane	NADH	Aramaki et al., 1993
1.14.13.162	2,5-diketocamphane 1,2-monooxygenase (CamE_25−1_, CamE_25−2_, CamE_36_)	*Pseudomonas putida* (ATCC 17453)	2,5-diketocamphane	Oxygen, NADH	(+)-5-oxo-1,2-campholide	Water, NAD^+^	Taylor and Trudgill, 1986
1.14.13.162	3,6-diketocamphane 1,6-monooxygenase (CamE_36_)	*Pseudomonas putida* (ATCC 17453)	3,6-diketocamphane	Oxygen, NADH	(−)-5-oxo-1,2-campholide	Water, NAD^+^	Taylor and Trudgill, 1986
6.2.1.38	(2,2,3-trimethyl-5-oxocyclopent-3-enyl) acetyl-CoA synthase (CamF1, CamF2)	*Pseudomonas putida* (ATCC 17453)	[(1*R*)-2,2,3-trimethyl-5-oxocyclopent-3-enyl] acetate	ATP, CoA	[(1*R*)-2,2,3-trimethyl-5-oxocyclopent-3-enyl] acetyl-CoA	Diphosphate, AMP	Ougham et al., 1983
1.14.13.160	2-oxo-Δ ^3^-4,5,5-trimethylcyclopentenyl acetyl-CoA 1,2-monooxygenase (CamG)	*Pseudomonas putida* (ATCC 17453)	[(1*R*)-2,2,3-trimethyl-5-oxocyclopent-3-enyl] acetyl-CoA	Oxygen, NADPH	[(2*R*)-3,3,4-trimethyl-6-oxo-3,6-dihydro-1H-pyran-2-yl] acetyl-CoA	Water, NADP^+^	Ougham et al., 1983; Leisch et al., 2012

## Bicyclic monoterpenes

**Figure 2:**
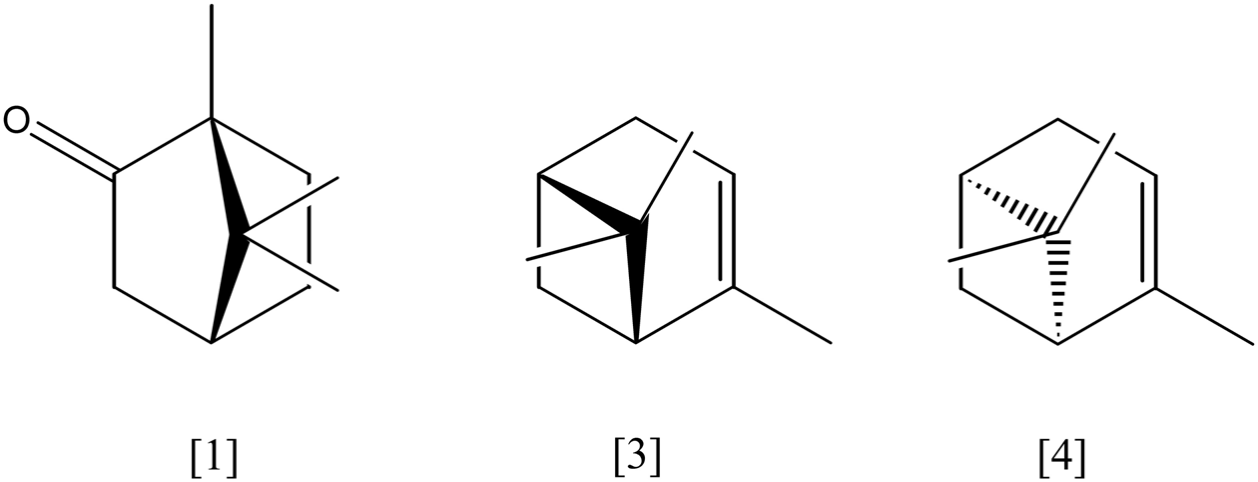
**(+)-camphor [1]; α-pinene [3]; β-pinene [4]**.

(+)-Camphor [**1**, Figure 2] (C_10_H_16_O) is the substrate of one of the first and best described monoterpene transforming enzymes, a specific cytochrome P450 monooxygenase (*camABC*, P450cam, EC 1.14.15.1) from *Pseudomonas putida* (ATCC 17453). Initially, (+)-camphor is hydroxylated. The resulting 5-*exo*-hydroxycamphor [**2**] is oxidized by a NAD-reducing dehydrogenase (EC 1.1.1.327) which gene *camD* is part of the operon *camDCAB*. The diketone is oxidized in a Baeyer–Villiger like oxidation to a lactone, either by a 2,5-diketocamphane 1,2-monooxygenase or a 3,6-diketocamphane 1,6-monooxygenase (*camE*_25−1_*E*_25−2_ or *camE*_36_, EC 1.14.13.162). The lactone spontaneously hydrolyses to 2-oxo-Δ^3^-4,5,5-trimethylcyclopentenyl-acetic acid which is activated as coenzyme A thioester by a specific synthase (*camF*_1,2_, EC 6.2.1.38). This CoA-ester serves as substrate for another specific monooxygenase (*camG*, EC 1.14.13.160), which initiates the cleavage of the second ring by formation of a lactone. After hydrolysis of the lactone, the linear product is oxidized to isobutanoyl-CoA and three acetyl-CoA. All corresponding genes (*camABCDEFG*) have been identified on a linear plasmid (Ougham et al., 1983; Taylor and Trudgill, 1986; Aramaki et al., 1993; Kadow et al., 2012; Leisch et al., 2012; Iwaki et al., 2013).

The most abundant bicyclic monoterpene is pinene with the isomers α-pinene [**3**] and β-pinene [**4**] (C_10_H_16_), a main constituent of wood resins (e.g. conifers). *Pseudomonas rhodesiae* (CIP 107491) and *P. fluorescens* (NCIMB 11671) grew on α-pinene as sole carbon source. α-pinene is oxidized to α-pinene oxide [**5**] by a NADH-dependent α-pinene oxygenase (EC 1.14.12.155) and undergoes ring cleavage by action of a specific α-pinene oxide lyase (EC 5.5.1.10), forming apparently isonovalal as first product which is isomerized to novalal (Best et al., 1987; Bicas et al., 2008; Linares et al., 2009). The cleavage reaction of α-pinene oxide was also described for a *Nocardia sp*. strain P18.3 (Griffiths et al., 1987; Trudgill, 1990, 1994).

An alternative route for pinene degradation via a monocyclic *p*-menthene derivate has been described for *Pseudomonas* sp. strain PIN (Yoo and Day, 2002). *Bacillus pallidus* BR425 degrades α- and β-pinene apparently via limonene [**6**] and pinocarveol. While α-pinene is transformed into limonene and pinocarveol, β-pinene yields pinocarveol only. Both intermediates may be further transformed into carveol **[7]** and carvone. The activity of a specific monooxygenases has been suggested, but experimental evidence is lacking (Savithiry et al., 1998). *Serratia marcescens* uses α-pinene as sole carbon source. *Trans*-verbenol **[8]** was a detectable metabolite. In glucose and nitrogen supplemented medium, this strain formed α-terpineol [**9**]. The two oxidation products were considered to be dead-end products as they accumulated in cultures (Wright et al., 1986). A general precaution has to be mentioned here for many biotransformation studies: monoterpenes contain often impurities and oxidation products which may be utilized as substrates resulting in traces of monoterpene and monoterpenoid transformation products that are not further metabolized. Stoichiometric experiments have to show that the amount of metabolite is larger than the amount of impurity in the substrate. Only such careful stoichiometric experiments, mutants in functional genes or the characterization of enzymes *in vitro* can provide a proof of the presence of a biotransformation.

Eucalyptol, the bicyclic monoterpene 1,8-cineole [**10**] (C_10_H_18_O), is transformed in several pathways. *Novosphingobium subterranea* converts 1,8-cineole initially into 2-*endo*-hydroxycineole, 2,2-oxo-cineole, and 2-*exo*-hydroxycineole. Acidic products from ring cleavages have been identified *in situ* (Rasmussen et al., 2005). Hydroxy-cineole formation occurred in 1,8-cineole-grown cultures of *Pseudomonas flava* (Carman et al., 1986). A cytochrome P450 monooxygenase from *Bacillus cereus* UI-1477 catalyzes the hydroxylation of 1,8-cineole, yielding either 2*R*-*endo*- or 2*R*-*exo*-hydroxy-1,8-cineole [**11**] (Liu and Rosazza, 1990, 1993). Another 1,8-cineole-specific P450 monooxygenase (EC 1.14.13.156) has been purified and characterized from *Citrobacter braakii*, which yielded 2-*endo*-hydroxy-1,8-cineole only. Further oxidation and lactonization were followed by a spontaneous lactone ring hydrolysis (Hawkes et al., 2002). Biotransformation in *Rhodococcus* sp. C1 involves an initial hydroxylation to 6-*endo*-hydroxycineol **[12]** and further oxidation to 6-oxocineole by a 6-*endo*-hydroxycineol dehydrogenase (EC 1.1.1.241). A 6-oxocineole monooxygenase (EC 1.14.13.51) converts the ketone into an unstable lactone. Spontaneous decomposition results in (*R*)-5,5-dimethyl-4-(3′-oxobutyl)-4,5-dihydrofuran-2(3H)-one. An initial monooxygenase activity has not been detected in cell-free systems, while the dehydrogenase and oxygenase activities have been measured in crude cell extracts (Williams et al., 1989).

## Monocyclic monoterpenes

**Figure 3:**
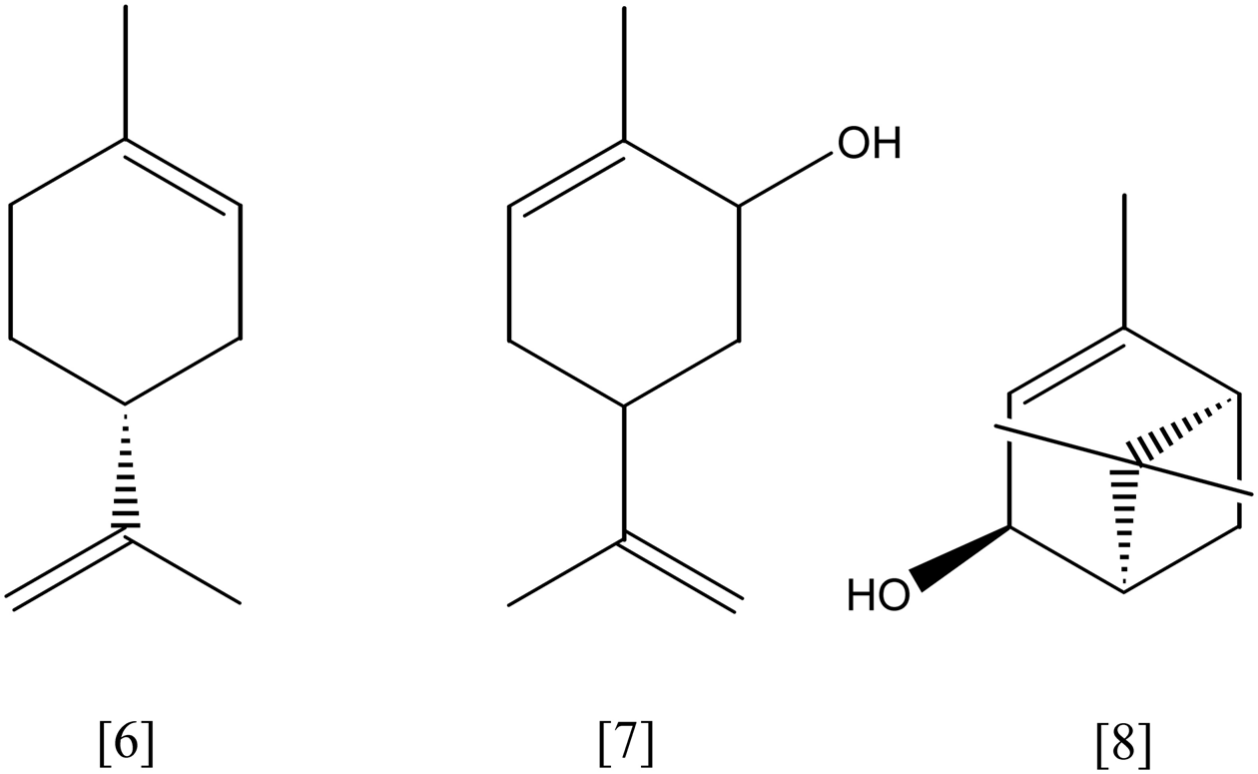
**(*R*)-limonene [6]; carveol [7]; *trans*-verbenol [8]**.

**Figure 5:**
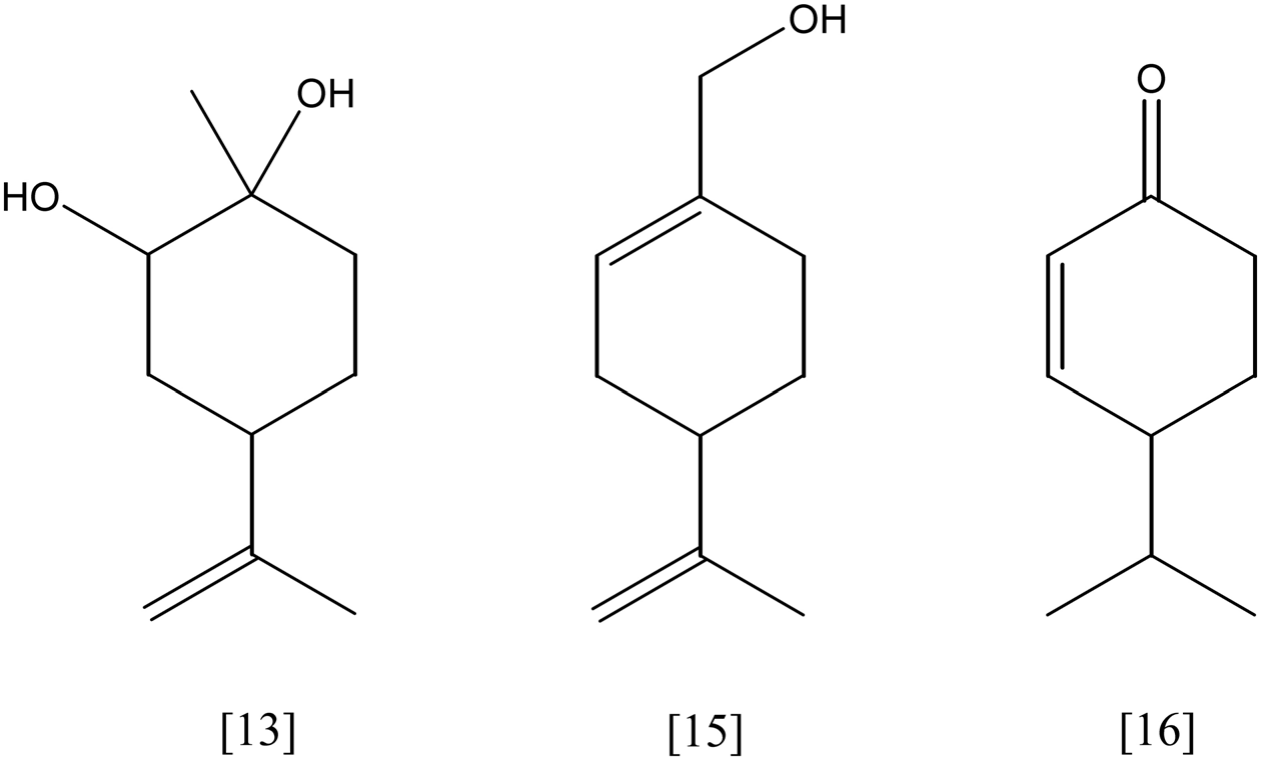
**limonene-1,2-diol [13]; perillyl alcohol [15]; cryptone [16]**.

**Figure 6:**
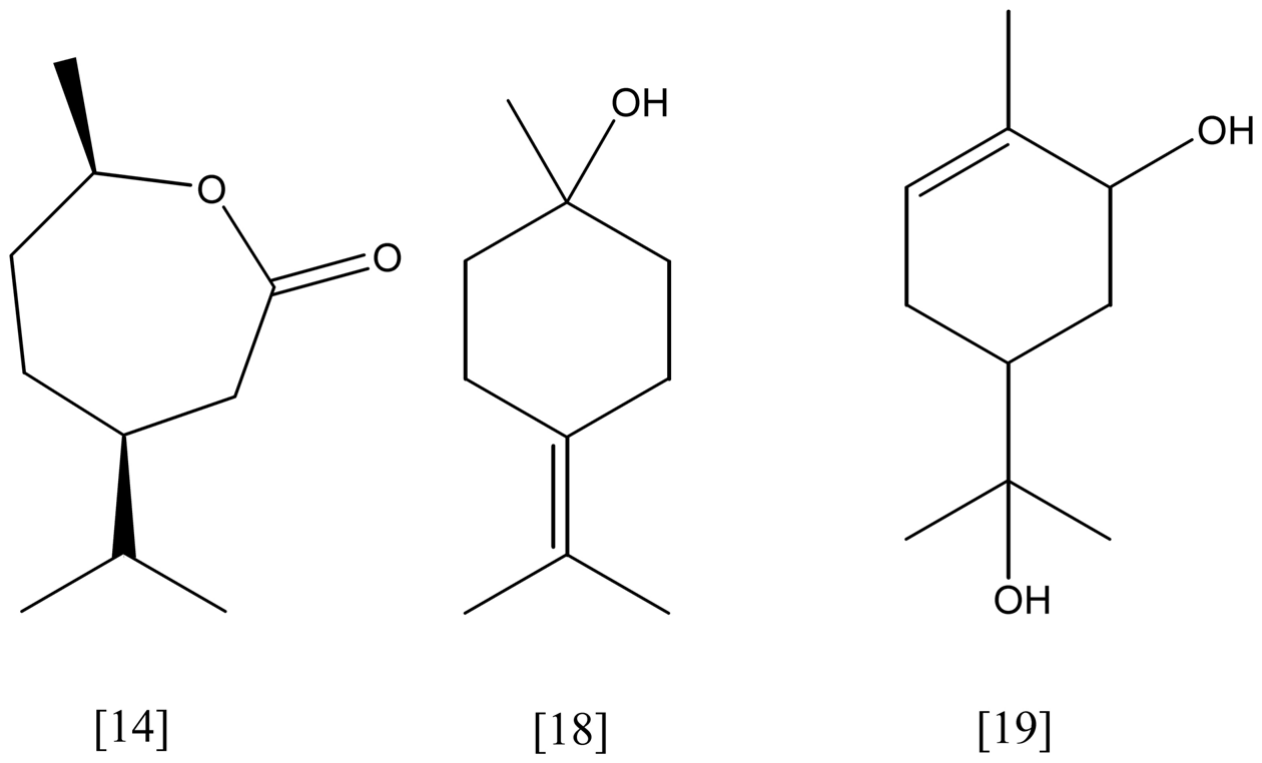
**isopropenyl-7-methyl-2-oxo-oxepanone [14]; γ-terpineol [18]; *p*-menth-1-ene-6,8-diol [19]**.

Limonene [**6**, Figure 3] (C_10_H_16_) is the most abundant monocyclic monoterpene, besides toluene the second most abundant VOC indoors (Brown et al., 1994). It represents the main component of essential oils from citrus plants, e.g. lemon and orange. *Rhodococcus erythropolis* DCL14 transforms (*R*/*S*)-limonene via limonene-1,2-epoxide into limonene-1,2-diol [**13**, Figure 5], applying a limonene-1,2 monooxygenase (EC 1.14.13.107) and a limonene-1,2-epoxide hydrolase (EC 3.3.2.8), respectively. A specific dehydrogenase (EC 1.1.1.297) forms the ketone, 1-hydroxy-2-oxolimonene, which is oxidized to a lactone by a 1-hydroxy-2-oxolimonene 1,2-monooxygenase (EC 1.14.13.105). Enzyme activities were only detected in limonene-induced cells, suggesting a tight regulation of the limonene degradation. *R. erythropolis* DCL14 harbors a second pathway for limonene degradation. Initially, (*R*)-limonene is hydroxylated by a NADPH-dependent limonene 6-monooxygenase (EC 1.14.13.48) to *trans*-carveol **[7]**. Subsequently, *trans*-carveol is oxidized to carvone and dihydrocarvone by a carveol dehydrogenase (EC 1.1.1.243) and carvone reductase (EC 1.3.99.25), respectively. A monocyclic monoterpene ketone monooxygenase (EC 1.14.13.105) inserts an oxygen atom, forming isopropenyl-7-methyl-2-oxo-oxepanone [14, Figure 6]. This lactone is cleaved by a specific ε-lactone hydrolase (EC 3.1.1.83) yielding hydroxyl-3-isopropenyl-heptanoate. Oxidation and activation as coenzyme A thioester enable a further degradation in accordance to the beta-oxidation (van der Werf et al., 1999b; van der Werf and Boot, 2000). *R. opacus* PWD4 uses (*R*)-limonene on the same pathway. Biomass from a glucose-toluene chemostat culture transformed limonene into *trans*-carveol, which was further oxidized to carvone by a *trans*-carveol dehydrogenase (EC 1.1.1.275) (Duetz et al., 2001).

**Figure 4:**
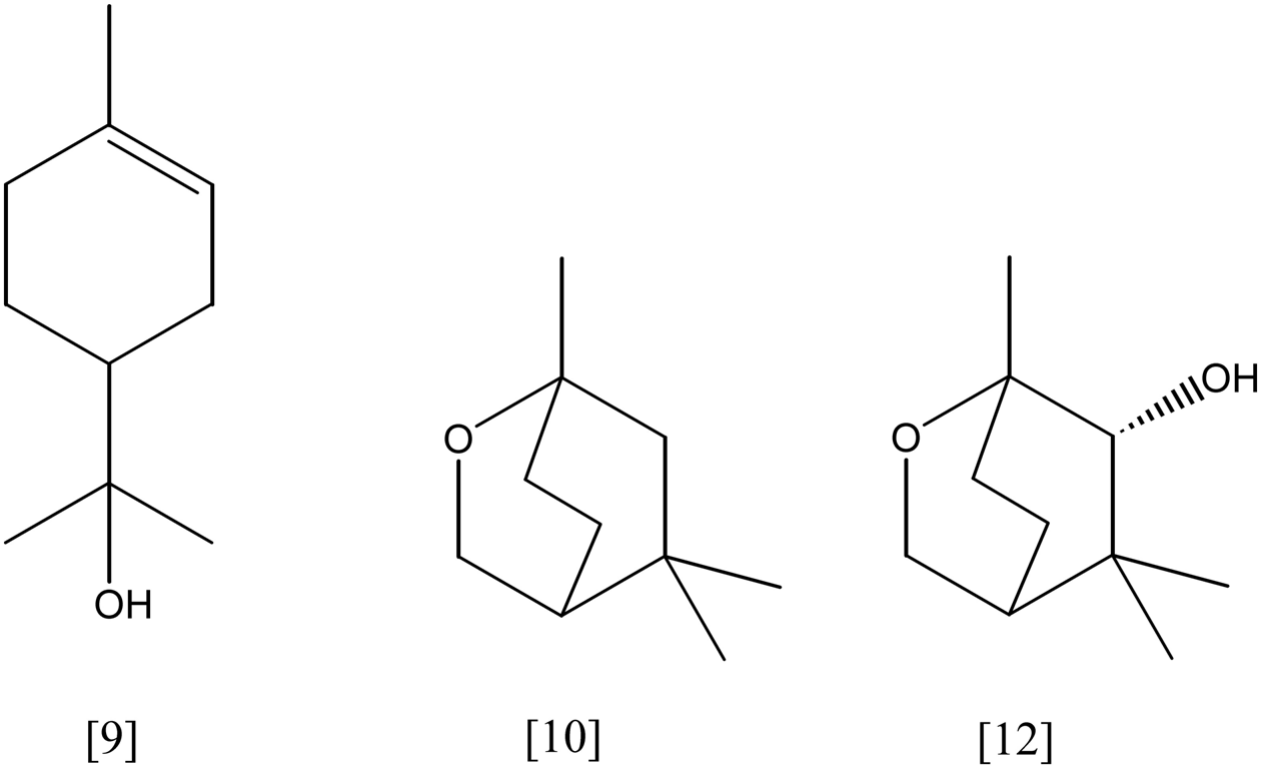
**α-terpineol [9]; 1,8-cineole [10]; 6-hydroxycineol [12]**.

Studies on the limonene metabolism in *P. gladioli* identified α-terpineol [**9**, Figure 4] and perillyl alcohol [**15**] as major metabolites. However, none of the involved enzymes has been purified or further characterized (Cadwallader et al., 1989). A α-terpineol dehydratase from *P. gladioli* was isolated and partially purified. The hydration reaction to the isopropenyl double bond of (4*R*)-(+)-limonene resulted in (4*R*)-(+)-α-terpineol as only product (Cadwallader et al., 1992). *Geobacillus stearothermophilus* (ex *Bacillus*) showed growth on limonene as sole carbon source. The main limonene transformation product was perillyl alcohol, while α-terpineol and perillyl aldehyde were found in minor concentrations. After heterologous expression of a putative limonene degradation pathway in *E. coli*, α-terpineol was identified as major product of the biotransformation. Other studies reported a limonene hydroxylation on the methyl group yielding perillyl alcohol, which underwent further oxidation to perillic acid (Chang and Oriel, 1994; Chang et al., 1995). Additional studies on the recombinant limonene hydroxylase confirmed the production of perillyl alcohol from limonene but revealed in addition the formation of carveol. The limonene hydroxylase showed dependency on molecular oxygen and NADH as cofactors and was suggested to belong to the (*S*)-limonene 7-monooxygenase family (EC 1.14.13.49) (Cheong and Oriel, 2000).

**Figure 8:**
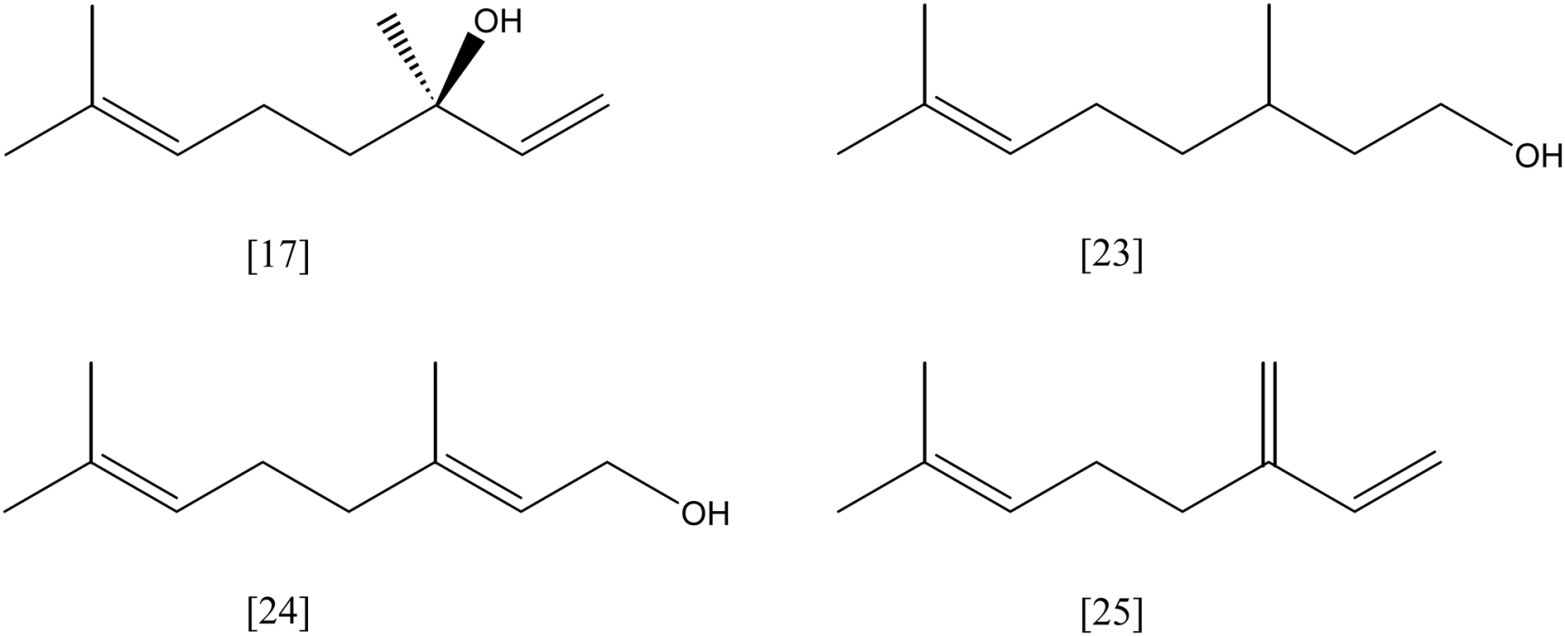
**(*S*)-(+)-linalool [17]; citronellol [23]; geraniol [24]; β-myrcene [25]**.

*Enterobacter agglomerans* 6L and *Kosakonia cowanii* 6L (ex *Enterobacter cowanii*) transformed (*R*)-limonene **[6]**. The main metabolites detected in ether extracts of *E. agglomerans* 6L cultures were γ-valerolactone and cryptone [**16**]. In assays using four recombinant expressed limonene-transforming enzymes from *K. cowanii* 6L, linalool [**17**, Figure 8] was identified as main product besides smaller amounts of dihydrolinalool. It was proposed that the potential limonene hydroxylase converts limonene into linalool, perillyl alcohol, α-terpineol and γ-terpineol [**18**] (Park et al., 2003; Yang et al., 2007).

*Pseudomonas putida* (MTCC 1072) converts limonene to *p*-menth-1-ene-6,8-diol **[19]** and perillyl alcohol (Chatterjee and Bhattacharyya, 2001). No sequence information was found in public databases. Two other strains of *Pseudomonas putida* (F1 and GS1) have been found to convert (+)-limonene to perillic acid in co-substrate fed-batch cultures (Speelmans et al., 1998). Experimental results indicated the participation of the *p*-cymene pathway (CYM) (Mars et al., 2001). *Castellaniella defragrans* grows anaerobically on cyclic monoterpenes as sole carbon and energy source under denitrifying conditions (Foss et al., 1998). Recent experiments suggested an oxygen-independent hydroxylation on the methyl group of limonene to perillyl alcohol as the initial activation step, followed by subsequent oxidation to perillic acid (Petasch et al., 2014).

**Figure 7:**
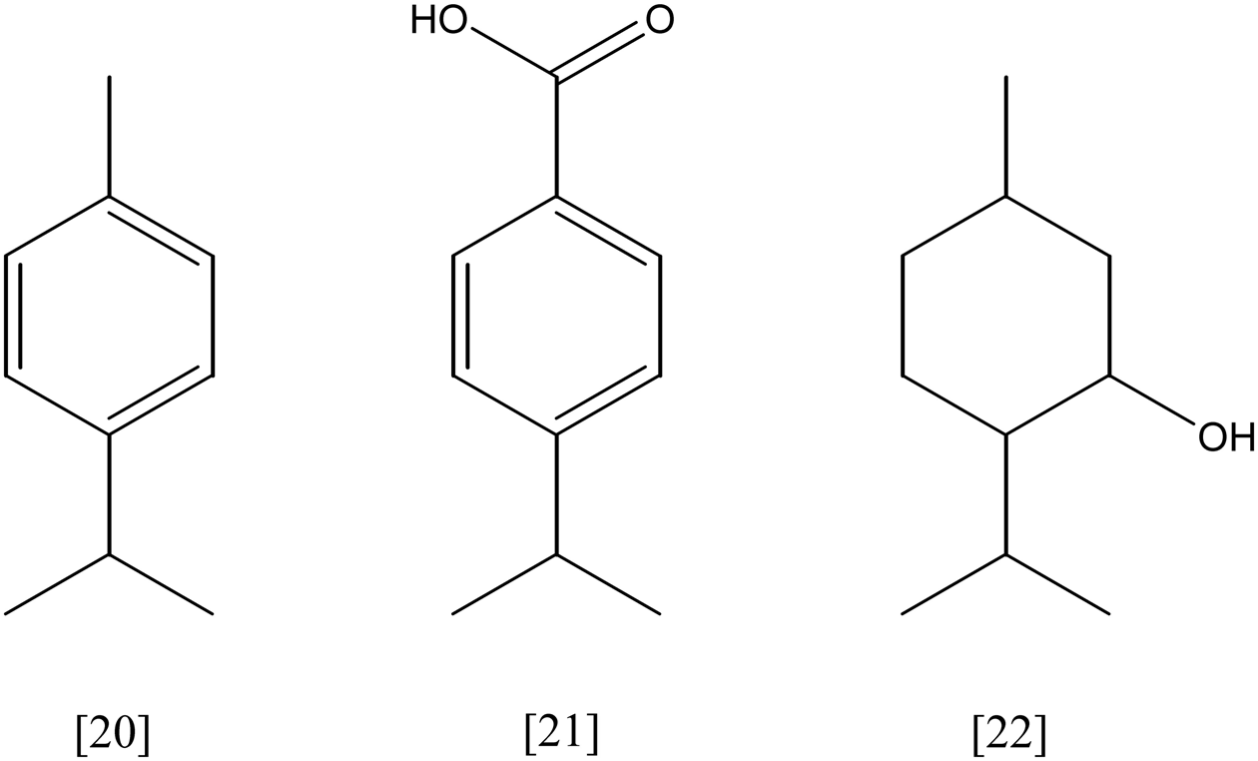
***p*-cymene [20]; *p*-cumate [21]; menthol [22]**.

*P*-cymene [**20**, Figure 7] (C_10_H_14_) is an aromatic monoterpene (*p*-isopropyl-toluene). *Pseudomonas putida* F1 (ATCC 700007) degrades *p*-cymene to *p*-cumate **[21]** via the CYM-pathway (*cymBCAaAbDE*). A two-component *p*-cymene monooxygenase (*cymAaAb*, EC 1.14.13.-) introduces a hydroxyl group on the methyl group of *p*-cymene. The resulting *p*-cumic alcohol is oxidized to the corresponding carboxylic acid by an alcohol and an aldehyde dehydrogenase (*cymB* and *cymC*, EC 1.1.1.- and EC 1.2.1.-). The genes *cymD* and *cymE* encode for a putative outer membrane protein and an acetyl coenzyme A synthetase, respectively. However, their role in the pathway remains unclear (Eaton, 1997). Upstream of the *cym*-operon, the genes for the further degradation of *p*-cumate are located. They are organized in another operon and comprise eight genes (*cmtABCDEFGH*). *P. putida* F1 has been shown to use *p*-cumate as sole carbon source. It is hydroxylated by a ferredoxin dependent *p*-cumate 2,3-dioxygenase. The genes *cmtAaAd* encode a ferredoxin reductase and a ferredoxin, and *cmtAbAc* encode the large and the small subunits of the dioxygenase (EC 1.14.12.-). The resulting *cis*-2,3-dihydroxy-2,3-dihydro-*p*-cumate is oxidized and ring cleavage occurs by introduction of another oxygen molecule. The responsible enzymes are a specific dehydrogenase (*cmtB*, EC 1.3.1.58) and a 2,3-dihydroxy-*p*-cumate dioxygenase (*cmtC*, EC 1.13.11.-), respectively. Further degradation is accomplished by a decarboxylation and elimination of an isobutyrate molecule, catalyzed by a 2-hydroxy-3-carboxy-6-oxo-7-methylocta-2,4-dienoate decarboxylase (*cmtD*, EC 4.1.1.-) and a 2-hydroxy-6-oxo-7-methylocta-2,4-dienoate hydrolase (*cmtE*, EC 3.7.1.-). The product, 2-hydroxypenta-2,4-dienoate, undergoes a water addition by a specific hydratase (*cmtF*, EC 4.2.1.80). Then, a carbon-carbon lyase reaction yields pyruvate and acetaldehyde, catalyzed by 2-oxo-4-hydroxyvalerate aldolase (*cmtG*, EC 4.1.3.39). Acetaldehyde is oxidized and enters as acetyl-CoA the citrate cycle (Eaton, 1996).

*Thauera terpenica* 21 Mol utilizes menthol **[22]** as sole carbon source. The proposed degradation mechanism involves two initial oxidation reactions leading to menth-2-enone, followed by a hydration and an additional oxidation step. Finally, ring cleavage may occur and the molecule is attached to coenzyme A to yield 3,7-dimethyl-5-oxo-octyl-CoA (Foss and Harder, 1998; Hylemon and Harder, 1998).

## Acyclic monoterpenes

First studies on acyclic monoterpenoids in the early sixties by Seubert and colleagues described the degradation of citronellol [**23**], geraniol [**24**], and nerol via an oxidation of the alcohol to an acid, followed by the formation of a CoA-thioester and subsequent beta-oxidation in *Pseudomonas citronellolis* (ATCC 13674) (Seubert, 1960; Seubert and Remberger, 1963; Seubert et al., 1963; Seubert and Fass, 1964). This knowledge has been extended toward other *Pseudomonas* strains (Cantwell et al., 1978). The complete degradation pathway has been classified as the acyclic terpene utilization and leucine utilization (ATU/LIU) pathway involving the genes *atuABCDEFGH* and *liuRABCDE*. After the initial formation of *cis*-geranyl-CoA, a geranyl-coenzyme-A carboxylase (*atuCF*, EC 6.4.1.5) elongates the methylgroup. A hydroxyl group is introduced by an isohexenyl-glutaconyl-CoA hydratase (*atuE*, EC 4.2.1.57), followed by a water addition and elimination of an acetate molecule catalyzed by a 3-hydroxy-3-isohexenylglutaryl-CoA lyase (*liu*E, EC 4.1.3.26). The resulting 7-methyl-3-oxooct-6-enoyl-CoA is further degraded via two beta-oxidation like reactions to yield 3-methylcrotonyl-CoA, which enters the leucine degradation pathway (*liuRABCDE*) (Höschle et al., 2005; Aguilar et al., 2006; Förster-Fromme et al., 2006; Chávez-Avilés et al., 2010; Förster-Fromme and Jendrossek, 2010). Citronellol degradation is reported for many *Pseudomonas* strains, including *P. aeruginosa* PAO1 (ATCC 15692), *P. mendocina* (ATCC 25411), and *P. delhiensis* (DSM 18900) (Cantwell et al., 1978; Prakash et al., 2007; Förster-Fromme and Jendrossek, 2010). Among the few reactions described in detail is a molybdenum dependent dehydrogenase responsible for the geranial oxidation to geranylate in *P. aeruginosa* PAO1 (Höschle and Jendrossek, 2005).

The acyclic monoterpene β-myrcene [**25**] (C_10_H_16_) is transformed by *Pseudomonas aeruginosa* (PTCC 1074) into dihydrolinalool, 2,6-dimethyloctane and α-terpineol. Limonene has been proposed as possible intermediate in α-terpineol formation but was not detected in the culture broth (Esmaeili and Hashemi, 2011). *Pseudomonas* sp. M1 accomplishes degradation by hydroxylation on the C8 position to myrcene-8-ol, which is further oxidized, linked to coenzyme A and metabolized in a beta-oxidation like manner (Iurescia et al., 1999). The formation of geraniol from β-myrcene has been observed with resting cells of *Rhodococcus erythropolis* MLT1, regardless of the presence of a cytochrome P450 inhibitor. The reaction was dependent on aerobic conditions, however it remains unclear if a monooxygenase or lyase system is involved (Thompson et al., 2010).

The tertiary alcohol linalool is also transformed at the C8 position. A linalool monooxygenase (EC 1.14.13.151) has been described in *P. putida* PpG777 and *Novosphingobium aromaticivorans* (ATCC 700278D-5) (Ullah et al., 1990; Bell et al., 2010). In the absence of molecular oxygen, *Castellaniella defragrans* 65Phen has an unique enzyme for the linalool transformation, the linalool dehydratase-isomerase (Brodkorb et al., 2010). *Castellaniella* and *Thauera* strains were the first anaerobic microorganisms shown to anaerobically degrade and mineralize monoterpenes (Harder and Probian, 1995; Harder et al., 2000). The linalool dehydratase-isomerase (EC 4.2.1.127 and 5.4.4.4) of *C. defragrans* 65Phen catalyzes a regio- and stereo-specific hydration of β-myrcene yielding the tertiary alcohol (*S*)-(+)-linalool [**17**] and the isomerization to the primary alcohol geraniol (Brodkorb et al., 2010; Lueddeke and Harder, 2011). Geraniol and geranial dehydrogenases formed geranic acid (Heyen and Harder, 2000; Lueddeke et al., 2012). *T. linaloolentis* 47Lol grows on linalool as sole carbon and energy source. A similar isomerization of linalool to geraniol with subsequent oxidation of geraniol to geranial has been observed in cultures (Foss and Harder, 1997).

## Monoterpene transformation by fungi

Fungi excrete laccases which are copper-containing oxidases. Utilizing molecular oxygen as a cosubstrate, an unspecific oxidation of organic molecules is initiated by these enzymes. Additionally, fungi express a variety of cytochrome P450 mono- and di-oxygenases. Thus, several fungi were described to transform monoterpenes during growth in rich medium (reviewed by Farooq et al., 2004). Species with a reported capacity to transform monoterpenes are *Aspergillus niger, Botrytis cinerea, Diplodia gossypina, Mucor circinelloides, Penicillium italicum, Penicillium digitatum, Corynespora cassiicola*, and *Glomerella cingulata*. For a long time, no species have been described to use monoterpenes as sole carbon and energy source for growth (Trudgill, 1994 and references therein). Recently, *Grosmannia clavigera*, a bark beetle-associated fungal pathogen of pine trees, was shown to grow on a mono- and diterpene mixture, containing α/β-pinene and 3-carene (Diguistini et al., 2011). ABC efflux transporter and cytochrome P450 enzymes confer a monoterpene resistance to the blue-stain fungi (Lah et al., 2013; Wang et al., 2013).

## Monoterpenes in the carbon cycle

Habitats with a dense vegetation of wood and flowers are expected to contain larger populations of monoterpene transforming microorganisms. Whereas coniferous forests emit up to 6.7 g carbon^*^m^−2*^yr^−1^, broadleaf evergreen forest and grassland emit only 3.5 and 2.5 g carbon^*^m^−2*^yr^−1^, respectively (Tanaka et al., 2012). Monoterpene emission rates between 0.3 and 7 g carbon^*^m^−2*^yr^−1^ for the United States—mainly α- and β-pinene, limonene and β-myrcene (Geron et al., 2000)—can support the aerobic growth of 0.15–3.5 g bacteria^*^m^−2*^yr^−1^, assuming 50% of carbon incorporated into biomass. This is a significant potential, considering the presence of around 10 g microbial biomass in the top centimeter of soil per square meter.

In marine systems, isoprene and monoterpenes (mainly α-pinene) are produced by phytoplankton and algae and partially emitted into the atmosphere (reviewed by Yassaa et al., 2008; Shaw et al., 2010). Isoprene emission was estimated to 0.2–1.2 Tg carbon^*^yr^−1^ (Palmer and Shaw, 2005; Gantt et al., 2009; Shaw et al., 2010). For the ocean surface area this results in an emission rate of 0.0025 g carbon^*^m^−2*^yr^−1^. Current uncertainties in the size of emission based on shipborne measurements in comparison to satellite data (Luo and Yu, 2010) may be resolved by incorporating an export from the continental atmosphere to the oceanic atmosphere (Hu et al., 2013). Isoprene-amended samples from marine habitats were enriched in bacteria affiliating with *Actinobacteria*, *Alphaproteobacteria*, and *Bacteroidetes* and first strains were shown to degrade isoprene and aliphatic hydrocarbons (Acuña Alvarez et al., 2009).

In summary, these findings indicate a higher abundance of monoterpene transforming and mineralizing bacteria in soils than in the ocean. Indeed, most monoterpene transforming bacteria have been enriched or isolated from soil and freshwater samples in habitats with monoterpene emitting vegetation.

## Databases for pathway analysis and a look at metagenomes

Databases are nowadays available for the analysis of enzymatic reactions and metabolic pathways in metagenomic and genomic sequence datasets. The most relevant are the Kyoto Encyclopedia of Genes and Genomes (KEGG), MetaCyc and the Biocatalysis/Biodegradation database of the University of Minnesota.

First studies used KEGG to identify monoterpene-related genes in metagenomes of microbiomes in insects and nematodes feeding on a monoterpene-rich diet. Pine beetles encounter the high terpenoid concentrations of conifers and may take advantage of detoxification processes catalyzed by their symbionts/microbiomes (Adams et al., 2013). The KEGG pathway for limonene and pinene degradation (ko00903) was used to identify genes encoding enzymes putatively involved in monoterpene degradation. Five enzymes were present and more abundant in the metagenomes than in a combined metagenomic set of plant biomass-degrading communities. These enzymes were an aldehyde dehydrogenase, an oxidoreductase, an enoyl-CoA hydratase and two hydratases/epimerases. Whether these genes are truly involved in monoterpene metabolism or the degradation of cyclic compounds, e.g. related aromatic lignin monomers, is an open question. Taxonomically, these genes affiliated with the genera *Pseudomonas*, *Rahnella*, *Serratia*, and *Stenotrophomonas*.

The pinewood nematode *Bursaphlenchus xylophilus* transcribes cytochrome P450 genes as main metabolic pathway for xenobiotics detoxification, but not all enzymes needed for terpenoid metabolism were detected by transcriptomic analysis. Metagenomic data of nematode bacterial symbionts included the complete α-pinene degradation pathway (Cheng et al., 2013). Annotation based on KEGG revealed that the degradation pathways for limonene and pinene (map00903) and for geraniol (map00281) accounted for 2.5% of mapped metagenes. The majority of these genes affiliated to *Pseudomonas*, *Achromobacter*, and *Agrobacterium*. Strains isolated from the nematode and capable of growth on α-pinene affiliated to *Pseudomonas*, *Achromobacter*, *Agrobacterium*, *Cytophaga*, *Herbaspirillum*, and *Stenotrophomonas*.

## Conclusion

The synthesis and transformation of BVOCs, especially terpenoids, by plants is well studied (Kesselmeier and Staudt, 1999). Corresponding pathways have been elucidated and a variety of corresponding enzymes have been isolated and characterized (Mahmoud and Croteau, 2002; Yu and Utsumi, 2009). In contrast, the exploration of the microbial transformation and mineralization of monoterpenes has accumulated a small coverage of the field. Simply, over the last 50 years, research on bacterial monoterpene metabolism had only found the interest of very few principal investigators. Now, large sequence datasets of organisms and biological communities provide an unprecedented insight into the diversity of pathways and provide us with challenging hypotheses. However, the basis for the annotation is the biochemical characterization of enzymes which is only available for few monoterpenes. Only three pathways are completely known on the genetic and enzymatic level: the ones for camphor (CAM), *p*-cymene (CYM/CMT), and citronellol/geraniol (ATU/LIU). For pinene, the gene for a key enzyme, the α-pinene oxide lyase (EC 5.5.1.10), is still unknown. The lack of such a key enzyme sequence for a KEGG pathway (map00903) illustrates our uncertainty in the interpretation of metagenomic and genomic datasets. Progress in proteomic and metabolomic analyses in the last years support now biochemical and genetic experiments which will swiftly reveal the desired identification of key enzymes in the monoterpene metabolism.

### Conflict of interest statement

The authors declare that the research was conducted in the absence of any commercial or financial relationships that could be construed as a potential conflict of interest.

## References

[B1] Acuña Alvarez L. Exton D. A. Timmis K. N. Suggett D. J. McGenity T. J. (2009). Characterization of marine isoprene-degrading communities. Environ. Microbiol. 11, 3280–3291 10.1111/j.1462-2920.2009.02069.x 19807779

[B2] Adams A. S. Aylward F. O. Adams S. M. Erbilgin N. Aukema B. H. Currie C. R. (2013). Mountain pine beetles colonizing historical and native host trees are associated with a bacterial community highly enriched in genes contributing to terpene metabolism. Appl. Environ. Microbiol. 79, 3468–3475 10.1128/AEM.00068-13 23542624 PMC3648045

[B3] Aguilar J. A. Zavala A. N. Díaz-Pérez C. Cervantes C. Díaz-Pérez A. L. Campos-García J. (2006). The *atu* and *liu* clusters are involved in the catabolic pathways for acyclic monoterpenes and leucine in *Pseudomonas aeruginosa*. Appl. Environ. Microbiol. 72, 2070–2079 10.1128/AEM.72.3.2070-2079.2006 16517656 PMC1393232

[B4] Ajikumar P. K. Tyo K. Carlsen S. Mucha O. Phon T. H. Stephanopoulos G. (2008). Terpenoids: opportunities for biosynthesis of natural product drugs using engineered microorganisms. Mol. Pharm. 5, 167–190 10.1021/mp700151b 18355030

[B5] Altman T. Travers M. Kothari A. Caspi R. Karp P. D. (2013). A systematic comparison of the MetaCyc and KEGG pathway databases. BMC Bioinformatics 14:112 10.1186/1471-2105-14-112 23530693 PMC3665663

[B6] Aramaki H. Koga H. Sagara Y. Hosoi M. Horiuchi T. (1993). Complete nucleotide-sequence of the 5-exo-hydroxycamphor dehydrogenase gene on the CAM plasmid of *Pseudomonas putida* (ATCC-17453). Biochim. Biophys. Acta 1174, 91–94 10.1016/0167-4781(93)90098-X 8334169

[B7] Atkinson R. Arey J. (2003). Atmospheric degradation of volatile organic compounds. Chem. Rev. 103, 4605–4638 10.1021/cr0206420 14664626

[B8] Bakkali F. Averbeck S. Averbeck D. Waomar M. (2008). Biological effects of essential oils - a review. Food Chem. Toxicol. 46, 446–475 10.1016/j.fct.2007.09.106 17996351

[B9] Bell S. G. Dale A. Rees N. H. Wong L. L. (2010). A cytochrome P450 class I electron transfer system from *Novosphingobium aromaticivorans*. Appl. Microbiol. Biotechnol. 86, 163–175 10.1007/s00253-009-2234-y 19779713

[B10] Best D. J. Floyd N. C. Magalhaes A. Burfield A. Rhodes P. M. (1987). Initial enzymatic steps in the degradation of alpha-pinene by *Pseudomonas fluorescens* Ncimb 11671. Biocatal. Biotransfor. 1, 147–159 10.3109/10242428709040139

[B11] Bicas J. L. Dionisio A. P. Pastore G. M. (2009). Bio-oxidation of terpenes: an approach for the flavor industry. Chem. Rev. 109, 4518–4531 10.1021/cr800190y 19645444

[B12] Bicas J. L. Fontanille P. Pastore G. M. Larroche C. (2008). Characterization of monoterpene biotransformation in two pseudomonads. J. Appl. Microbiol. 105, 1991–2001 10.1111/j.1365-2672.2008.03923.x 19120646

[B13] Brodkorb D. Gottschall M. Marmulla R. Lueddeke F. Harder J. (2010). Linalool dehydratase-isomerase, a bifunctional enzyme in the anaerobic degradation of monoterpenes. J. Biol. Chem. 285, 30436–30442 10.1074/jbc.M109.084244 20663876 PMC2945536

[B14] Brown S. K. Sim M. R. Abramson M. J. Gray C. N. (1994). Concentrations of volatile organic compounds in indoor air - a review. Indoor Air 4, 123–134

[B15] Cadwallader K. R. Braddock R. J. Parish M. E. (1992). Isolation of alpha-terpineol dehydratase from *Pseudomonas gladioli*. J. Food Sci. 57, 241 10.1111/j.1365-2621.1992.tb05464.x

[B16] Cadwallader K. R. Braddock R. J. Parish M. E. Higgins D. P. (1989). Bioconversion of (+)-limonene by *Pseudomonas gladioli*. J. Food Sci. 54, 1241–1245 10.1111/j.1365-2621.1989.tb05964.x

[B17] Cantwell S. G. Lau E. P. Watt D. S. Fall R. R. (1978). Biodegradation of acyclic isoprenoids by *Pseudomonas* species. J. Bacteriol. 135, 324–333 681275 10.1128/jb.135.2.324-333.1978 PMC222387

[B18] Carman R. M. Macrae I. C. Perkins M. V. (1986). The oxidation of 1,8-cineole by *Pseudomonas flava*. Aust. J. Chem. 39, 1739–1746 10.1071/CH9861739

[B19] Chang H. C. Gage D. A. Oriel P. J. (1995). Cloning and expression of a limonene degradation pathway from *Bacillus stearothermophilus* in *Escherichia coli*. J. Food Sci. 60, 551–553 10.1111/j.1365-2621.1995.tb09824.x

[B20] Chang H. C. Oriel P. (1994). Bioproduction of perillyl alcohol and related monoterpenes by isolates of *Bacillus stearothermophilus*. J. Food Sci. 59, 660 10.1111/j.1365-2621.1994.tb05588.x

[B21] Chatterjee T. Bhattacharyya D. K. (2001). Biotransformation of limonene by *Pseudomonas putida*. Appl. Microbiol. Biotechnol. 55, 541–546 10.1007/s002530000538 11414318

[B22] Chávez-Avilés M. Díaz-Pérez A. L. Campos-García J. (2010). The bifunctional role of LiuE from *Pseudomonas aeruginosa*, displays additionally HIHG-CoA lyase enzymatic activity. Mol. Biol. Rep. 37, 1787–1791 10.1007/s11033-009-9611-6 19597963

[B23] Cheng X. Y. Tian X. L. Wang Y. S. Lin R. M. Mao Z. C. Chen N. S. (2013). Metagenomic analysis of the pinewood nematode microbiome reveals a symbiotic relationship critical for xenobiotics degradation. Sci. Rep. 3:1869 10.1038/srep01869 23694939 PMC3660777

[B24] Cheong T. K. Oriel P. J. (2000). Cloning and expression of the limonene hydroxylase of *Bacillus stearothermophilus* BR388 and utilization in two-phase limonene conversions. Appl. Biochem. Biotechnol. 84–86, 903–915 10.1385/ABAB:84-86:1-9:903 10849845

[B25] Dhavalikar R. Bhattacharyya P. (1966). Microbiological transformation of terpenes. 8. Fermentation of limonene by a soil *Pseudomonad*. Indian J. Biochem. 3, 144–157 4227570

[B26] Dhavalikar R. Rangachari P. Bhattacharyya P. (1966). Microbiological transformations of terpenes. 9. Pathways of degradation of limonene in a soil *Pseudomonad*. Indian J. Biochem. 3, 158–164 4227571

[B27] Díaz-Pérez A. L. Zavala-Hernandez A. N. Cervantes C. Campos-Garcia J. (2004). The gnyRDBHAL cluster is involved in acyclic isoprenoid degradation in *Pseudomonas aeruginosa*. Appl. Environ. Microbiol. 70, 5102–5110 10.1128/AEM.70.9.5102-5110.2004 15345388 PMC520886

[B28] Diguistini S. Wang Y. Liao N. Y. Taylor G. Tanguay P. Feau N. (2011). Genome and transcriptome analyses of the mountain pine beetle-fungal symbiont *Grosmannia clavigera*, a lodgepole pine pathogen. Proc. Natl. Acad. Sci. U.S.A. 108, 2504–2509 10.1073/pnas.1011289108 21262841 PMC3038703

[B29] Dudareva N. Klempien A. Muhlemann J. K. Kaplan I. (2013). Biosynthesis, function and metabolic engineering of plant volatile organic compounds. New Phytol. 198, 16–32 10.1111/nph.12145 23383981

[B30] Duetz W. A. Bouwmeester H. Van Beilen J. B. Witholt B. (2003). Biotransformation of limonene by bacteria, fungi, yeasts, and plants. Appl. Microbiol. Biotechnol. 61, 269–277 10.1007/s00253-003-1221-y 12743755

[B31] Duetz W. A. Fjallman A. H. M. Ren S. Y. Jourdat C. Witholt B. (2001). Biotransformation of D-limonene to (+) trans-carveol by toluene-grown *Rhodococcus opacus* PWD4 cells. Appl. Environ. Microbiol. 67, 2829–2832 10.1128/AEM.67.6.2829-2832.2001 11375201 PMC92945

[B32] Eaton R. W. (1996). *p*-Cumate catabolic pathway in *Pseudomonas putida* F1: cloning and characterization of DNA carrying the *cmt* operon. J. Bacteriol. 178, 1351–1362 8631713 10.1128/jb.178.5.1351-1362.1996 PMC177810

[B33] Eaton R. W. (1997). *p*-Cymene catabolic pathway in *Pseudomonas putida* F1: cloning and characterization of DNA encoding conversion of *p*-cymene to *p*-cumate. J. Bacteriol. 179, 3171–3180 9150211 10.1128/jb.179.10.3171-3180.1997 PMC179094

[B34] Esmaeili A. Hashemi E. (2011). Biotransformation of myrcene by *Pseudomonas aeruginosa*. Chem. Cent. J. 5:26 10.1186/1752-153X-5-26 21609445 PMC3127812

[B35] Fall R. R. Hector M. L. (1977). Acyl-coenzyme-a carboxylases. Homologous 3-methylcrotonyl-coA and geranyl-coA carboxylases from *Pseudomonas citronellolis*. Biochemistry 16, 4000–4005 911753 10.1021/bi00637a010

[B36] Farooq A. Atta-Ur-Rahman Choudhary M. I. (2004). Fungal transformation of monoterpenes. Curr. Org. Chem. 8, 353–367 10.2174/1385272043485945

[B37] Fontanille P. Le Flèche A. Larroche C. (2002). *Pseudomonas rhodesiae* PF1: a new and efficient biocatalyst for production of isonovalal from α-pinene oxide. Biocatal. Biotransformation 20, 413–421 10.1080/1024242021000058702

[B38] Förster-Fromme K. Höschle B. Mack C. Bott M. Armbruster W. Jendrossek D. (2006). Identification of genes and proteins necessary for catabolism of acyclic terpenes and leucine/isovalerate in *Pseudomonas aeruginosa*. Appl. Environ. Microbiol. 72, 4819–4828 10.1128/AEM.00853-06 16820476 PMC1489323

[B39] Förster-Fromme K. Jendrossek D. (2010). Catabolism of citronellol and related acyclic terpenoids in pseudomonads. Appl. Microbiol. Biotechnol. 87, 859–869 10.1007/s00253-010-2644-x 20490788

[B40] Foss S. Harder J. (1997). Microbial transformation of a tertiary allylalcohol: regioselective isomerisation of linalool to geraniol without nerol formation. FEMS Microbiol. Lett. 149, 71–75 10.1016/S0378-1097(97)00057-8

[B41] Foss S. Harder J. (1998). *Thauera linaloolentis* sp. nov. and *Thauera terpenica* sp. nov., isolated on oxygen-containing monoterpenes (linalool, menthol, and eucalyptol) and nitrate. Syst. Appl. Microbiol. 21, 365–373 10.1016/S0723-2020(98)80046-5 9841126

[B42] Foss S. Heyen U. Harder J. (1998). *Alcaligenes defragrans* sp. nov., description of four strains isolated on alkenoic monoterpenes ((+)-menthene, alpha-pinene, 2-carene, and alpha-phellandrene) and nitrate. Syst. Appl. Microbiol. 21, 237–244 10.1016/S0723-2020(98)80028-3 9704110

[B43] Fu P. Q. Kawamura K. Chen J. Barrie L. A. (2009). Isoprene, monoterpene, and sesquiterpene oxidation products in the high arctic aerosols during late winter to early summer. Environ. Sci. Technol. 43, 4022–4028 10.1021/es803669a 19569325

[B44] Gantt B. Meskhidze N. Kamykowski D. (2009). A new physically-based quantification of marine isoprene and primary organic aerosol emissions. Atmos. Chem. Phys. 9, 4915–4927 10.5194/acp-9-4915-2009

[B45] Geron C. Rasmussen R. Arnts R. R. Guenther A. (2000). A review and synthesis of monoterpene speciation from forests in the United States. Atmos. Environ. 34, 1761–1781 10.1016/S1352-2310(99)00364-7

[B46] Ghirardo A. Gutknecht J. Zimmer I. Bruggemann N. Schnitzler J. P. (2011). Biogenic volatile organic compound and respiratory CO_2_ emissions after ^13^C-labeling: online tracing of C translocation dynamics in poplar plants. PLoS ONE 6:e17393 10.1371/journal.pone.0017393 21387007 PMC3046154

[B47] Griffiths E. T. Harries P. C. Jeffcoat R. Trudgill P. W. (1987). Purification and properties of alpha-pinene oxide lyase from *Nocardia sp*. strain P18.3. J. Bacteriol. 169, 4980–4983 3667522 10.1128/jb.169.11.4980-4983.1987 PMC213896

[B48] Guenther A. Hewitt C. N. Erickson D. Fall R. Geron C. Graedel T. (1995). A global model of natural volatile organic compound emissions. J. Geophys. Res. Atmos. 100, 8873–8892 10.1029/94JD02950

[B49] Harayama S. Rekik M. Ngai K. L. Ornston L. N. (1989). Physically associated enzymes produce and metabolize 2-hydroxy-2,4-dienoate, a chemically unstable intermediate formed in catechol metabolism via meta-cleavage in *Pseudomonas putida*. J. Bacteriol. 171, 6251–6258 2681159 10.1128/jb.171.11.6251-6258.1989 PMC210496

[B50] Harder J. Foss S. (1999). Anaerobic formation of the aromatic hydrocarbon *p*-cymene from monoterpenes by methanogenic enrichment cultures. Geomicrobiol. J. 16, 295–305 10.1080/014904599270550

[B51] Harder J. Heyen U. Probian C. Foss S. (2000). Anaerobic utilization of essential oils by denitrifying bacteria. Biodegradation 11, 55–63 10.1023/A:1026552724696 11194974

[B52] Harder J. Probian C. (1995). Microbial degradation of monoterpenes in the absence of molecular oxygen. Appl. Environ. Microbiol. 61, 3804–3808 8526489 10.1128/aem.61.11.3804-3808.1995 PMC167682

[B53] Hawkes D. B. Adams G. W. Burlingame A. L. De Montellano P. R. O. De Voss J. J. (2002). Cytochrome P450_cin_ (CYP176A), isolation, expression, and characterization. J. Biol. Chem. 277, 27725–27732 10.1074/jbc.M203382200 12016226

[B54] Heyen U. Harder J. (2000). Geranic acid formation, an initial reaction of anaerobic monoterpene metabolism in denitrifying *Alcaligenes defragrans*. Appl. Environ. Microbiol. 66, 3004–3009 10.1128/AEM.66.7.3004-3009.2000 10877798 PMC92103

[B55] Höschle B. Gnau V. Jendrossek D. (2005). Methylcrotonyl-CoA and geranyl-CoA carboxylases are involved in leucine/isovalerate utilization (Liu) and acyclic terpene utilization (Atu), and are encoded by *liuB*/*liuD* and *atuC*/*atuF*, in *Pseudomonas aeruginosa*. Microbiology 151(pt 11), 3649–3656 10.1099/mic.0.28260-0 16272386

[B56] Höschle B. Jendrossek D. (2005). Utilization of geraniol is dependent on molybdenum in *Pseudomonas aeruginosa*: evidence for different metabolic routes for oxidation of geraniol and citronellol. Microbiology 151, 2277–2283 10.1099/mic.0.27957-0 16000717

[B57] Hu Q. H. Xie Z. Q. Wang X. M. Kang H. He Q. F. Zhang P. F. (2013). Secondary organic aerosols over oceans via oxidation of isoprene and monoterpenes from Arctic to Antarctic. Sci. Rep. 3:2280 10.1038/srep02280 23880782 PMC3721125

[B58] Hylemon P. B. Harder J. (1998). Biotransformation of monoterpenes, bile acids, and other isoprenoids in anaerobic ecosystems. FEMS Microbiol. Rev. 22, 475–488 10.1111/j.1574-6976.1998.tb00382.x 9990726

[B59] Insam H. Seewald M. S. A. (2010). Volatile organic compounds (VOCs) in soils. Biol. Fertil. Soils 46, 199–213 10.1007/s00374-010-0442-3

[B60] Ishida T. (2005). Biotransformation of terpenoids by mammals, microorganisms, and plant-cultured cells. Chem. Biodivers. 2, 569–590 10.1002/cbdv.200590038 17192005

[B61] Iurescia S. Marconi A. M. Tofani D. Gambacorta A. Paterno A. Devirgiliis C. (1999). Identification and sequencing of beta-myrcene catabolism genes from *Pseudomonas sp.* strain M1. Appl. Environ. Microbiol. 65, 2871–2876 10388678 10.1128/aem.65.7.2871-2876.1999 PMC91431

[B62] Iwaki H. Grosse S. Bergeron H. Leisch H. Morley K. Hasegawa Y. (2013). Camphor pathway redux: functional recombinant expression of 2,5- and 3,6-diketocamphane monooxygenases of *Pseudomonas putida* ATCC 17453 with their cognate flavin reductase catalyzing Baeyer-Villiger reactions. Appl. Environ. Microbiol. 79, 3282–3293 10.1128/AEM.03958-12 23524667 PMC3685261

[B63] Kadow M. Loschinski K. Sass S. Schmidt M. Bornscheuer U. T. (2012). Completing the series of BVMOs involved in camphor metabolism of *Pseudomonas putida* NCIMB 10007 by identification of the two missing genes, their functional expression in *E. coli*, and biochemical characterization. Appl. Microbiol. Biotechnol. 96, 419–429 10.1007/s00253-011-3859-1 22286514

[B64] Kainulainen P. Holopainen J. K. (2002). Concentrations of secondary compounds in scots pine needles at different stages of decomposition. Soil Biol. Biochem. 34, 37–42 10.1016/S0038-0717(01)00147-X

[B65] Kesselmeier J. Staudt M. (1999). Biogenic volatile organic compounds (VOC): an overview on emission, physiology and ecology. J. Atmos. Chem. 33, 23–88 10.1023/A:1006127516791

[B66] Lah L. Haridas S. Bohlmann J. Breuil C. (2013). The cytochromes P450 of *Grosmannia clavigera*: genome organization, phylogeny, and expression in response to pine host chemicals. Fungal Genet. Biol. 50, 72–81 10.1016/j.fgb.2012.10.002 23111002

[B67] Leisch H. Shi R. Grosse S. Morley K. Bergeron H. Cygler M. (2012). Cloning, Baeyer-Villiger biooxidations, and structures of the camphor pathway 2-oxo-Δ^3^-4,5,5-trimethylcyclopentenylacetyl-coenzyme A monooxygenase of *Pseudomonas putida* ATCC 17453. Appl. Environ. Microbiol. 78, 2200–2212 10.1128/AEM.07694-11 22267661 PMC3302634

[B68] Li H. J. Lan W. J. (2011). Biotransformation of limonene by microorganisms. Prog. Chem. 23, 2318–2325

[B69] Li H. J. Lan W. J. Cai C. H. Zhou Y. P. Lin Y. C. (2006). Biotransformation of limonene by marine bacteria. Chin. J. Anal. Chem. 34, 946–950 10.1016/S1872-2040(06)60046-7

[B70] Linares D. Fontanille P. Larroche C. (2009). Exploration of A-pinene degradation pathway of *Pseudomonas rhodesiae* cip 107491. Application to novalic acid production in a bioreactor. Food Res. Int. 42, 461–469 10.1016/j.foodres.2008.12.001

[B71] Liu W. G. Rosazza J. P. N. (1990). Stereospecific hydroxylation of 1,8-cineole using a microbial biocatalyst. Tetrahedron Lett. 31, 2833–2836 10.1016/0040-4039(90)80160-N

[B72] Liu W. G. Rosazza J. P. N. (1993). A soluble *Bacillus cereus* cytochrome-P-450_cin_ system catalyzes 1,4-cineole hydroxylations. Appl. Environ. Microbiol. 59, 3889–3893 8285692 10.1128/aem.59.11.3889-3893.1993 PMC182545

[B73] Lueddeke F. Harder J. (2011). Enantiospecific (*S*)-(+)-linalool formation from beta-myrcene by linalool dehydratase-isomerase. Z. Naturforsch. C 66, 409–412 10.5560/ZNC.2011.66c0409 21950166

[B74] Lueddeke F. Wuelfing A. Timke M. Germer F. Weber J. Dikfidan A. (2012). Geraniol and geranial dehydrogenases induced in anaerobic monoterpene degradation by *Castellaniella defragrans*. Appl. Environ. Microbiol. 78, 2128–2136 10.1128/AEM.07226-11 22286981 PMC3302621

[B75] Luo G. Yu F. (2010). A numerical evaluation of global oceanic emissions of α-pinene and isoprene. Atmos. Chem. Phys. 10, 2007–2015 10.5194/acp-10-2007-2010

[B76] Mahmoud S. S. Croteau R. B. (2002). Strategies for transgenic manipulation of monoterpene biosynthesis in plants. Trends Plant Sci. 7, 366–373 10.1016/S1360-1385(02)02303-8 12167332

[B77] Mars A. E. Gorissen J. P. L. Van Den Beld I. Eggink G. (2001). Bioconversion of limonene to increased concentrations of perillic acid by *Pseudomonas putida* GS1 in a fed-batch reactor. Appl. Microbiol. Biotechnol. 56, 101–107 10.1007/s002530100625 11499915

[B78] Martinez J. L. Sánchez M. B. Martínez-Solano L. Hernandez A. Garmendia L. Fajardo A. (2009). Functional role of bacterial multidrug efflux pumps in microbial natural ecosystems. FEMS Microbiol. Rev. 33, 430–449 10.1111/j.1574-6976.2008.00157.x 19207745

[B79] Ougham H. J. Taylor D. G. Trudgill P. W. (1983). Camphor revisited - Involvement of a unique monooxygenase in metabolism of 2-oxo-Δ^3^-4,5,5-trimethylcyclopentenylacetic acid by *Pseudomonas putida*. J. Bacteriol. 153, 140–152 6848481 10.1128/jb.153.1.140-152.1983 PMC217351

[B80] Palmer P. I. Shaw S. L. (2005). Quantifying global marine isoprene fluxes using modis chlorophyll observations. Geophys. Res. Lett. 32, L09805 10.1029/2005GL022592

[B81] Papadopoulos C. J. Carson C. F. Chang B. J. Riley T. V. (2008). Role of the mexab-oprm efflux pump of *Pseudomonas aeruginosa* in tolerance to tea tree (*Melaleuca altenifolia*) oil and its monoterpene components terpinen-4-ol, 1,8-cineole, and alpha-terpineol. Appl. Environ. Microbiol. 74, 1932–1935 10.1128/AEM.02334-07 18192403 PMC2268291

[B82] Park Y. J. Kim I. C. Chang H. C. (2003). Microbial conversion of (+)-limonene by an *Enterobacter agglomerans* isolate. J. Microbiol. Biotechnol. 13, 636–639

[B118] Petasch J. Disch E.-M. Markert S. Becher D. Schweder T. Hüttel B. (2014). The oxygen-independent metabolism of cyclic monoterpenes in *Castellaniella defragrans* 65Phen. BMC Microbiol. 14:164 10.1186/1471-2180-14-164 24952578 PMC4109377

[B83] Platt A. Shingler V. Taylor S. C. Williams P. A. (1995). The 4-hydroxy-2-oxovalerate aldolase and acetaldehyde dehydrogenase (acylating) encoded by the *nahM* and *nahO* genes of the naphthalene catabolic plasmid pWW60-22 provide further evidence of conservation of meta-cleavage pathway gene sequences. Microbiology 141, 2223–2233 7496535 10.1099/13500872-141-9-2223

[B84] Poulos T. L. Finzel B. C. Gunsalus I. C. Wagner G. C. Kraut J. (1985). The 2.6-A crystal structure of *Pseudomonas putida* cytochrome-P-450. J. Biol. Chem. 260, 6122–6130 4066706

[B85] Prakash O. Kumari K. Lal R. (2007). *Pseudomonas delhiensis* sp. nov., from a fly ash dumping site of a thermal power plant. Int. J. Syst. Evol. Microbiol. 57, 527–531 10.1099/ijs.0.64456-0 17329778

[B86] Rasmussen J. A. M. Henderson K. A. Straffon M. J. Dumsday G. J. Coulton J. Zachariou M. (2005). Two new biocatalysts for improved biological oxidation of 1,8-cineole. Aust. J. Chem. 58, 912–916 10.1071/CH05204

[B87] Savithiry N. Gage D. Fu W. J. Oriel P. (1998). Degradation of pinene by *Bacillus pallidus* BR425. Biodegradation 9, 337–341 10.1023/A:1008304603734 10192895

[B88] Schewe H. Mirata M. A. Holtmann D. Schrader J. (2011). Biooxidation of monoterpenes with bacterial monooxygenases. Process Biochem. 46, 1885–1899 10.1016/j.procbio.2011.06.010

[B89] Seubert W. (1960). Degradation of isoprenoid compounds by microorganisms. i. isolation and characterization of an isoprenoid-degrading bacterium, *Pseudomonas citronellolis* n. sp. J. Bacteriol. 79, 426–434 14445211 10.1128/jb.79.3.426-434.1960 PMC278703

[B90] Seubert W. Fass E. (1964). Untersuchungen über den bakteriellen Abbau von Isoprenoiden. 5. Der Mechanismus des Isoprenabbaus. Biochem. Z. 341, 35–44 14339652

[B91] Seubert W. Fass E. Remberger U. (1963). Untersuchungen über den bakteriellen Abbau von Isoprenoiden. 3. Reinigung und Eigenschaften der Geranylcarboxylase. Biochem. Z. 338, 265–275 14087299

[B92] Seubert W. Remberger U. (1963). Untersuchungen über den bakteriellen Abbau von Isoprenoiden. 2. Die Rolle der Kohlensäure. Biochem. Z. 338, 245–264 14087298

[B93] Sharkey T. D. Wiberley A. E. Donohue A. R. (2008). Isoprene emission from plants: why and how. Ann. Bot. 101, 5–18 10.1093/aob/mcm240 17921528 PMC2701830

[B94] Sharkey T. D. Yeh S. S. (2001). Isoprene emission from plants. Annu. Rev. Plant Physiol. Plant Mol. Biol. 52, 407–436 10.1146/annurev.arplant.52.1.407 11337404

[B95] Shaw S. L. Gantt B. Meskhidze N. (2010). Production and emissions of marine isoprene and monoterpenes: a review. Adv. Meteorol. 2010:408696 10.1155/2010/408696

[B96] Speelmans G. Bijlsma A. Eggink G. (1998). Limonene bioconversion to high concentrations of a single and stable product, perillic acid, by a solvent-resistant *Pseudomonas putida* strain. Appl. Microbiol. Biotechnol. 50, 538–544 10.1007/s002530051331

[B97] Stotzky G. Schenck S. (1976). Volatile organic compounds and microorganisms. Crit. Rev. Microbiol. 4, 333–382 10.3109/10408417609102303 780055

[B98] Tanaka K. Kim H. J. Saito K. Takahashi H. G. Watanabe M. Yokohata T. (2012). How have both cultivation and warming influenced annual global isoprene and monoterpene emissions since the preindustrial era? Atmos. Chem. Phys. 12, 9703–9718 10.5194/acp-12-9703-2012

[B99] Taylor D. G. Trudgill P. W. (1986). Camphor revisited: studies of 2,5-diketocamphane 1,2-monooxygenase from *Pseudomonas putida* ATCC 17453. J. Bacteriol. 165, 489–497 3944058 10.1128/jb.165.2.489-497.1986 PMC214445

[B100] Thompson M. L. Marriott R. Dowle A. Grogan G. (2010). Biotransformation of beta-myrcene to geraniol by a strain of *Rhodococcus erythropolis* isolated by selective enrichment from hop plants. Appl. Microbiol. Biotechnol. 85, 721–730 10.1007/s00253-009-2182-6 19707757

[B101] Tong W.-Y. (2013). Biotransformation of terpenoids and steroids, in Natural Products, eds Ramawat K. G. Mérillon J.-M. (Berlin; Heidelberg: Springer), 2733–2759 10.1007/978-3-642-22144-6_122

[B102] Trudgill P. W. (1990). Microbial metabolism of monoterpenes - recent developments. Biodegradation 1, 93–105 10.1007/BF00058829 1368150

[B103] Trudgill P. W. (1994). Microbial Metabolism And Transformation Of Selected Monoterpenes. Dordrecht; Norwell: Kluwer Academic Publishers

[B104] Ullah A. J. H. Murray R. I. Bhattacharyya P. K. Wagner G. C. Gunsalus I. C. (1990). Protein components of a cytochrome P-450 linalool 8-methyl hydroxylase. J. Biol. Chem. 265, 1345–1351 2295633

[B105] van der Werf M. De Bont J. A. M. Leak D. J. (1997). Opportunities in microbial biotransformation of monoterpenes, in Advances In Biochemical Engineering Biotechnology; Biotechnology Of Aroma Compounds, ed Berger R. G. (Berlin; New York, NY: Springer-Verlag), 147–177

[B106] van der Werf M. J. Boot A. M. (2000). Metabolism of carveol and dihydrocarveol in *Rhodococcus erythropolis* DCL14. Microbiology 146, 1129–1141 10832640 10.1099/00221287-146-5-1129

[B107] van der Werf M. J. Orru R. V. A. Overkamp K. M. Swarts H. J. Osprian I. Steinreiber A. (1999a). Substrate specificity and stereospecificity of limonene-1,2-epoxide hydrolase from *Rhodococcus erythropolis* DCL14; an enzyme showing sequential and enantioconvergent substrate conversion. Appl. Microbiol. Biotechnol. 52, 380–385 9748436

[B108] van der Werf M. J. Swarts H. J. De Bont J. A. M. (1999b). *Rhodococcus erythropolis* DCL14 contains a novel degradation pathway for limonene. Appl. Environ. Microbiol. 65, 2092–2102 10224006 10.1128/aem.65.5.2092-2102.1999 PMC91303

[B109] Wang Y. Lim L. Diguistini S. Robertson G. Bohlmann J. Breuil C. (2013). A specialized ABC efflux transporter GcABC-G1 confers monoterpene resistance to *Grosmannia clavigera*, a bark beetle-associated fungal pathogen of pine trees. New Phytol. 197, 886–898 10.1111/nph.12063 23252416

[B110] Williams D. R. Trudgill P. W. Taylor D. G. (1989). Metabolism of 1,8-cineole by a *Rhodococcus* species: ring cleavage reactions. J. Gen. Microbiol. 135, 1957–1967

[B111] Wilt F. M. Miller G. C. Everett R. L. Hackett M. (1993). Monoterpene concentrations in fresh, senescent, and decaying foliage of single-leaf pinyon (*Pinus monophylla* Torr And Frem: Pinaceae) from the Western Great-Basin. J. Chem. Ecol. 19, 185–194 10.1007/BF00993688 24248867

[B112] Wright S. J. Caunt P. Carter D. Baker P. B. (1986). Microbial oxidation of α-pinene by *Serratia marcescens*. Appl. Microbiol. Biotechnol. 23, 224–227

[B113] Yang J. E. Park Y. J. Chang H. C. (2007). Cloning of four genes involved in limonene hydroxylation from *Enterobacter cowanii* 6L. J. Microbiol. Biotechnol. 17, 1169–1176 18051329

[B114] Yassaa N. Peeken I. Zollner E. Bluhm K. Arnold S. Spracklen D. (2008). Evidence for marine production of monoterpenes. Environ. Chem. 5, 391–401 10.1071/EN08047

[B115] Yoo S. K. Day D. F. (2002). Bacterial metabolism of alpha- and beta-pinene and related monoterpenes by *Pseudomonas* sp. strain PIN. Process Biochem. 37, 739–745 10.1016/S0032-9592(01)00262-X

[B116] Yu F. N. A. Utsumi R. (2009). Diversity, regulation, and genetic manipulation of plant mono- and sesquiterpenoid biosynthesis. Cell. Mol. Life Sci. 66, 3043–3052 10.1007/s00018-009-0066-7 19547916 PMC11115753

[B117] Ziemann P. J. Atkinson R. (2012). Kinetics, products, and mechanisms of secondary organic aerosol formation. Chem. Soc. Rev. 41, 6582–6605 10.1039/c2cs35122f 22940672

